# Process intensification for the protein production from microalgae: a comprehensive review

**DOI:** 10.1186/s40643-026-01118-0

**Published:** 2026-09-01

**Authors:** Arijit Nath, Abubakar Saleh Ahmad, Munkhnasan Enkhbold, Fatma Elzhraa, Gabriella Kiskó

**Affiliations:** 1 Department of Food Process Engineering, Institute of Food Science and Technology, Hungarian University of Agriculture and Life Sciences, Ménesi st 44, Budapest, HU-1118 Hungary; 2 International Center of Excellence in Seafood Science and Innovation, Faculty of Agro-Industry, Prince of Songkla University, 15 Kanchanawanich Road, Hat Yai, Songkhla, 90110 Thailand; 3 Department of Refrigeration and Livestock Product Technology, Institute of Food Science and Technology, Hungarian University of Agriculture and Life Sciences, Ménesi st 43-45,, Budapest, HU-1118 Hungary; 4 Department of Food Hygiene, Safety, and Technology, Faculty of Veterinary Medicine, Mansoura University, El-Gomhoria street, Mansoura, 35516 Egypt; 5 Department of Food Microbiology, Hygiene and Safety, Institute of Food Science and Technology, Hungarian University of Agriculture and Life Sciences, Somlói St 14-16, Budapest, HU-1118 Hungary

**Keywords:** Protein from microalgae, Mutant phenotype, Recombinant strain, Operational strategy of cultivation, Operating parameters, Process intensification

## Abstract

Microalgae (predominantly unicellular photosynthetic eukaryotes) has been recognized as a “protein bio-factory” because they may produce up to 70% of protein (dry basis). Advantageous aspects of proteins from microalgae compared to conventional animal- and plant-based proteins were proven. Numerous review articles have addressed the effects of composition of cultivation medium and operational parameters to obtain high protein yield from microalgae. Nevertheless, several critical limitations remain for the industrial expansions of the production of proteins from microalgae. Presently, process intensification is considered a strategic approach for the scalable production of proteins from microalgae, achieved by implementing mutagenic and recombinant strains, increasing productivity through high-cell-density cultivation strategies, using cold-adapted microalgae and minimizing equipment footprint via integrating multiple unit operations into a more efficient and compact system. Different strategies of random mutagenesis (chemical: ethyl methane sulfonate-mediated, physical: heavy-ion irradiation-mediated and ultraviolet-mediated) have been employed to get the desired phenotype of microalgae for higher protein yield. Emerging genetic engineering approaches, including the design of expression vectors, identification of genetic regulatory elements, and genetic transformation methods, along with bioinformatics algorithms, have been employed to develop recombinant strains of microalgae with higher protein yield. Combined cultivation systems, such as sequential heterotrophic-autotrophic system, switching of nitrogen-rich cultivation medium from nitrogen-deficient medium and mixotrophic system have come to the forefront. In this review article, process intensification strategies for the production of proteins from microalgae are comprehensively discussed, with special emphasis on the development of mutant and recombinant strains, strategies of cultivation and decisive operational parameters of bioreactor operation.

## Introduction

**Fig. 1:**
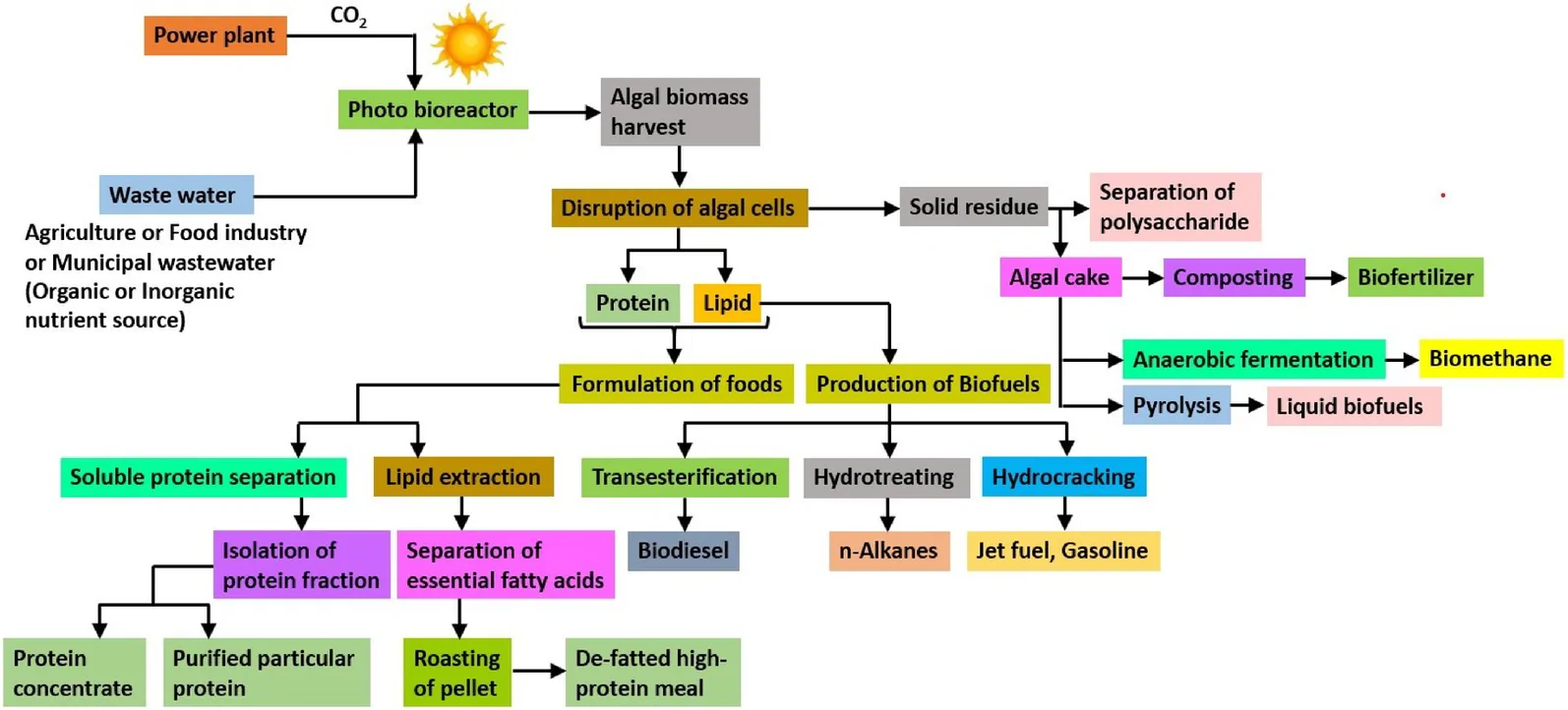
Nexus between water, energy and food systems through algal biotechnology. Algae are cultivated in photobioreactors, where agricultural or food industry or municipal wastewater can serve as nutrient sources, simultaneously supporting biomass production and wastewater remediation. During cultivation, microalgae assimilate CO₂ from the atmosphere and perform photosynthesis under sunlight, converting inorganic carbon (CO_2_) into organic macromolecules (polysaccharide, protein, lipid) through a cascade of metabolic reactions. This carbon assimilation process enhances biomass of microalgae while formation of valuable macronutrients. The biochemical process is known as photoautotrophic growth. Sunlight is not required for the heterotrophic growth of microalgae. In that mode of cultivation, the microalgae could use organic carbons from waste water as their energy sources instead of relying on sunlight for the photosynthesis. Furthermore, certain microalgae can grow under mixotrophic conditions by simultaneously utilizing sunlight and organic carbon sources. Macroalgae are primarily cultivated in natural or controlled aquatic environments and rely mainly on photoautotrophic growth. Microalgae generally contain lower amount of carbohydrate but are rich in proteins and lipids. Macroalgae are rich in soluble fibers and complex polysaccharides. Following sufficient biomass accumulation, algal cells are harvested, and intracellular metabolites, such as polysaccharides, lipids and proteins are extracted using wide ranges of techniques. The residual biomass can be utilized as a feedstock for the production of biofertilizer by composting, biomethane by anaerobic digestion and liquid biofuel by pyrolysis process. Remaining fractions rich in essential fatty acids, amino acids, and proteins are suitable for food and feed formulations (self-developed, concept was adopted from Zabochnicka et al. (2022)

Recently, the Food and Agriculture Organization (FAO) of the United Nations highlighted the nexus among water, energy and food to encourage resource conservation, food security and circular economy (Correa-Porcel et al. 2021). In this context, the biotransformation of atmospheric and aquatic CO_2_, and utilization of waste water through oxygenic photosynthesis by eukaryotic algae and cyanobacteria have opened numerous prospects (Lee, and Lavoie, 2013). Thereby, cultivation, harvesting, processing and exploring the opportunities associated with algae have opened a new horizon, collectively known as “algal biotechnology” (Fabris et al. 2020). Historically, attempts were considered for the commercial production of biofuels from algal biomass; however, their productions at any significant scale with economically viable sense were not achieved. Presently, focus has largely shifted towards the production of wide ranges of compounds and formulations having high importance in foods and biopharmaceutical industries (Khan et al. 2018). Algae can be considered as a “nutrient biofactory” and have opened a new age of “phycogastronomy” in which biological functionality and culinary modernization intersect (Nova et al. 2020; Torres-Tiji et al. 2020). Microalgae and macroalgae provide wide range of nutritional benefits because they are rich sources of proteins and their derivative peptides, soluble fibers, polysaccharides and their derivatives (alginate, carrageenans and agar), lipids (phospholipids, glycolipids, non-polar glycerolipids and unusual fatty acids), pigments (chlorophylls a, b and c, fucoxanthin, peridinin, phycobilins, phycoerythrin and phycocyanin), vitamins (vitamin A, vitamin B, vitamin C, vitamin D, vitamin E, vitamin K), and macro-and microelements (Ferreira de Oliveira and Bragotto 2022). Furthermore, algae can provide specific flavor, ranging from umami, grassy, salty and nutty to extend neutral (Matos et al. 2022). Owing to their rapid growth rates, high biomass productivity and ability to grow on non-arable land using wastewater, algae represent a sustainable resource for food, feed and energy generations. A process flow diagram of algal biotechnology in the context of 3rd generation of biofuels and resolving the issue related with food security and circular economy is presented in Fig. 1.

The current international Recommended Dietary Allowance (RDA) for protein is 0.8 g·kg of body weight^−1^, regardless of age (Wu 2016; Lonnie et al. 2018). According to the British nutrition foundation, the reference nutrient intake of protein for adults is 0.75 g of protein·kg body weight^−1^·day^−1^. It equates to 45 g of protein·day^−1^ and 56 g of protein·day^−1^ for women and men with average body weights 60 Kg and 75 Kg, respectively (Matos et al. 2022). In order to meet the urgent protein requirements due to the future insufficient protein supply, microalgae as a source of protein has recently grabbed attention (Soto-Sierra et al. 2018; Wang et al. 2021). The global protein market was valued at ~ 38 billion USD in 2019 and is projected to grow at a rate of 9.1% from 2020 to 2027 (Ismail et al. 2020). Aligned with it, the microalgae-based protein market is expected to rise from ~ 0.6 billion USD in 2018 to ~ 0.84 billion USD by 2023, reflecting increasing consumer interest (Delgado 2003). Although whole microalgae have been widely commercialized by both established and emerging companies for applications in foods, dietary supplements and food additives (Chen et al. 2022a), the commercialization of microalgal-derived proteins remains limited. The primary disadvantage of utilizing whole-cell microalgae instead of extracted microalgal proteins in food applications, is the lower protein digestibility caused by the presence of cell wall polysaccharides, including homopolysaccharides (α-rhamnans, β-galactofuranans, β-glucans, β-mannans and cellulose) and heteropolysaccharides (hemicellulose and pectin) and reserve polysaccharides (branched starches and β-glucans) (Van De Walle et al. 2024). Therefore, it may be realized that greater emphasis is needed to place to understand scalable and economically viable strategies for the production of protein from microalgae.

Numerous review articles have addressed the potentialities of protein production of different strains of microalgae. Furthermore, effects of different operational parameters, such as light intensity, wavelength, photoperiod, nutrient concentration, temperature, pH of cultivation medium and cultivation mode on microalgal growth and protein accumulation in laboratory- and bench-scale experimental setups; however, the industrial expansions of the production of proteins from microalgae have not meticulously practiced (Gao et al. 2024; Karabulut et al. 2024). Selection of the suitable strain of microalgae with higher growth rates and the potential for protein biosynthesis remain priorities for the industrial-scale protein production from microalgae. This selection process is closely associated with regulatory approval by the U.S. Food and Drug Administration (FDA) and the European Food Safety Authority (EFSA) for the implementation of microalgae in the food system regardless of their proven beneficiary nutritional aspects (Janssen et al. 2022; Cruz and Vasconcelos 2024). Furthermore, the selection of strain is closely associated with the choice of cultivation method, bioreactor configuration and nutrient bioavailability for the industrial-scale protein production from microalgae. Efficient dewatering and downstream processing techniques for the extraction of protein from microalgae are also considered crucial factors to satisfy the supply-chain demand (Grossmann et al. 2020; Novoveská et al. 2023). The process of protein production from microalgal could become sustainable and economically viable by addressing the aforementioned challenges.

Recent laboratory-based research outcomes indicate that process intensification is indispensable to bridge the gap between laboratory-scale research and industrial-scale production of microalgal proteins. Process intensification aims to enhance the protein productivity using mutagenic and recombinant strains while minimizing energy consumption, promoting by-product recycling and reducing the equipment footprint through integrating the principles of multiple unit operations into more efficient systems. The implementation of process intensification is essential for the commercial-scale production of proteins from microalgae because conventional processes often fail to achieve the protein productivity, resource utilization and economic feasibility essential for industrial-scale production. In the industrial-scale microalgal protein production scheme, minimizing energy consumption, cultivation time and the expenditure for downstream processing while maintaining reliable product quality is essential for commercial viability. Therefore, strategies related to process intensification provide a practical framework to overcome the technical and economic limitations associated with the scale-up of protein production from microalgae (Grützner et al. 2018). The integration of biological and engineering strategies represents a key framework for the process intensification of microalgal protein production. Process integration can improve the overall process efficiency by combining different steps of the bioprocess, including cultivation and harvesting of microalgae, and downstream processing of proteins, into streamlined production platforms. From biochemical and bioprocess engineering perspectives, process intensification strategies maximize the space-time yield by improving the amount of protein produced per unit reactor volume and cultivation time. Furthermore, process intensification facilitates the efficient utilization of nutrients (including carbon sources and water), thereby contributing to resource management and a sustainable ecosystem (Woodley 2017). Different approaches to intensify the bioprocess have been investigated to maximize protein yield and biomass productivity while minimizing resource consumption and production costs. For microalgal protein production, these approaches may include strain development, the use of cold-adapted microalgae, the development of high-cell-density cultivation strategies and by-product utilization (Sarkar et al. 2026). Therefore, the proper selection of biological and engineering strategies can increase volumetric productivity of proteins from microalgae and reduce cultivation time. The successful implementation of process intensification strategies requires a thorough understanding of microalgal physiology and its response to operational parameters. Without detailed knowledge of how light distribution, nutrient availability and stress conditions influence metabolic pathways related to protein biosynthesis, intensification efforts may lead to metabolic imbalances or reduced protein quality.

It can be expected that a comprehensive discussion of process intensification strategies for the production of protein from microalgae will promote its commercial production and help to meet supply chain demands. Realizing this fact, comprehensive information about the process intensification for the scalable production of proteins from microalgae with special emphasis on development of mutants and recombinant strains of microalgae, strategies of cultivation, using cold-adapted microalgae and decisive operational parameters of bioreactor operation (light spectrum, intensity and photoperiod, temperature, and the concentrations of inorganic and organic carbon sources, and nitrogen) is presented in this review article.

## Microalgae as a source of proteins

The biological functionalities of proteins are dependent on the quality of the protein and its building block (composition of amino acid: non-essential or essential, glucogenic or ketogenic and bioavailability of amino acids). However; the plant, animal and dairy-based proteins provide all types of amino acids and play a significant role in nutrition, a range of debatable issues have been coming to forefront. Presence of the high concentration of insoluble polysaccharides with plant-based proteins may cause the difficulty of its digestion (Ismail et al. 2020). Animal proteins are associated with saturated fats and cholesterol, which may increase the risks of hyper-cholesterolemia, cardiovascular diseases and diabetes (Zhubi-Bakija et al. 2021). Furthermore, presence of different types of epitopes (IgE- and IgG-binding) in conventional plant and animal-based proteins may cause their limitation of consumptions (Seidler et al. 2024).

Vegan, vegetarian and flexitarian consumers have different choices for selecting their dietary proteins. Considering all aforementioned issues, it may feel that microalgae could be a suitable candidate among other animal- and plant- based protein sources because microalgae provide higher amount of proteins (50–70%) compared to soybean (35%), chickpea (18%), milk (26%), turkey (63%) and beef (50%) (Soto-Sierra et al. 2018; Chen et al. 2022a), and protein from microalgae may be a suitable choice in vegetarian and vegan diet charts (Wang et al. 2021). While microalgae were first systematically considered as a food supplement in the 1950s, their applications as a single-cell protein for animal and human nutrition were introduced later in the 1970s (Soeder and Pabst 1970), with studies reporting species, such as *Scenedesmus* sp. as a potential single-cell protein source (Venkataraman et al. 1977). It was evident that protein in most of the species of microalgae is abundant (up to 70% by dry weight) than other macronutrients (lipids (4–20%) and carbohydrates (12–30%); however, these could vary according to species (Mosibo et al. 2024). Among all types of microalgae, *Spirulina*,* Chlorella*,* Dunaliella*,* Chlamydomonas* and *Scenedesmus* contain higher amount of proteins (50–70% weight basis) (Matos 2019) and nutritionally-favorable essential amino acid (EAA) profiles (Becker 2007).

Microalgae can provide all types of amino acids including 20 EAAs, similar to soybean, meat, egg and milk proteins (Gantar and Svirčev 2008). In microalgae, EAAs represent almost a half of total amino acids (Černá 2011), which meet the FAO recommended requirements (Bleakley and Hayes 2017). Proteins in microalgae have both glucogenic amino acids (proline, serine, aspartic acid, glycine, alanine, ornithine, glutamic acid, lysine and valine) and ketogenic amino acids (leucine, lysine, tryptophan, phenylalanine and tyrosine) (Zheng et al. 2025). Free amino acids (FAAs) are abundant in microalgae and it varies among different species of microalgae. Along with providing all types of EAAs, microalgal proteins are related to mimicking sensory aspects, including aroma, color, taste and textural mouthfeel (Isleten Hosoglu 2018). It has been reported that free glutamic acid and aspartic acid are mainly responsible for ‘umami’ sensation (Mouritsen et al. 2019); whereas, glycine and alanine are responsible for sweet flavor (Norziah and Ching 2000). Some other quality parameters of proteins from microalgae, such as amino acid score, EAA index, chemical score, biological value, protein digestibility (both in *vitro* and in *vivo*), net protein utilization, protein efficiency ratio, protein digestibility corrected amino acid score, digestibility coefficient and digestible indispensable amino acid score are comparable with conventional plant- and animal- proteins (Lucakova et al. 2022; Severo et al. 2024); however, these values could be reduced when whole cell of microalgae is considered for dietary intake.

Microalgae are considered valuable cell factories because they have ability to harvest light and convert CO_2_ to biomass and a variety of biomolecules, including proteins (photoautotrophic growth). They can also grow in waste water with a high organic load in the absence of light by utilizing organic carbon sources (heterotrophic growth). Therefore, microalgae can be cultivated in wastewater or non-potable water with minimal nutrient and energy inputs, which reduces the competition with edible plant and animal protein sources for arable land and freshwater resources (Norsker et al. 2011). Production of proteins from microalgae required lower land space compared to animal-based proteins. For example, per kg of protein production; <2.5 m^2^ is required for microalgae (Van et al. 2013); whereas, 144–258 m^2^ is required for beef, 47–64 m^2^ is required for pork and 42–52 m^2^ is required for chicken. Similarly, microalgae provide higher protein yield per unit area compared to terrestrial crops. For example, 4–15 tons·Ha^−1^·year^−1^ is for microalgae; whereas, 0.6–1.2 tons·Ha^−1^·year^−1^ is for leguminous soybean, 1–2 tons·Ha^−1^·year^−1^ is for pulse and 1.1 tons·Ha^−1^·year^−1^ is for wheat, respectively (de Vries and de Boer 2010).

Microalgae synthesize a wide range of proteins, including structural proteins, membrane proteins, metabolic enzymes, storage proteins and photosynthetic proteins. Some of these proteins possess highly nutritional importance owing to its well-balanced EAAs composition. Phycobiliproteins function as accessory pigments in the photosynthetic apparatus of cyanobacteria and red algae; whereas, most green microalgae (Chlorophyta) utilize chlorophyll-binding light-harvesting complex (LHC) proteins (Bleakley and Hayes 2017). Phycobiliproteins are classified into four major categories based on their absorption spectral properties, such as phycoerythrins (pink-purple; λ_max_ 540–570 nm), phycoerythrocyanins (orange; λ_max_ 560–600 nm), phycocyanins (blue; λ_max_ 610–620 nm) and allophycocyanins (blue-green; λ_max_ 650–655 nm) (Chen et al. 2022b). The techno-functionalities of proteins from microalgae play an important role in food processing apart from their nutritional value. These properties are dependent on the composition of amino acids of proteins and further it is influenced by the applied techniques for the extraction of proteins from microalgae (Nunes et al. 2024). Techno-functionalities of proteins from microalgae significantly influence (a) surface properties (solubility, emulsification and foaming), (b) hydrodynamic properties (gelation, viscosity, thickening and texturization) (Pan-utai and Iamtham 2023) and organoleptic properties of food products. Furthermore, several researches have demonstrated that algal protein-derived peptides have anti-oxidative, anti-inflammatory, anti-aging, anti-hypertensive, anti-tumor, anti-bacterial, anti-viral, osteogenic, neuroprotective and hepatoprotective activities (Harnedy and FitzGerald 2011).

## Process intensification for production of proteins from microalgae

The quality and content of proteins in microalgae depend on several factors, such as (a) type of microalgae, (b) genetic aspects of microalgae, (c) cultivation system of microalgae (d) the composition of cultivation medium of microalgae and (e) decisive operational parameters and strategies of bioreactor operation (Wang et al. 2021; Raja et al. 2022). To intensify the scalable production of proteins from microalgae, emphasis was placed on the development of mutants and recombinant strains, strategies of cultivation and decisive operational parameters of bioreactor operation. Different operational parameters, such as light spectrum, intensity and photoperiod, temperature and the concentrations of inorganic and organic carbon sources, and nitrogen were taken into consideration.

### Genetic modification of microalgae

Genetic modification and expression of heterologous genes in microalgae have emerged as important strategies for the enhancement of protein production by microalgae. Microalgae possess considerable potential as alternative protein sources; however, wild-type of microalgae often exhibits suboptimal biomass productivity, inadequate protein biosynthesis and insufficient robustness under large-scale cultivation conditions, which limits their commercialization (Kumar et al. 2020). Consequently, the development of improved strains of microalgae has received an attention in microalgal biotechnology. Advanced microalgae have been developed mainly for 3 aspects, such as high biomass yield through photosynthesis, production of protein and lipid, and production of precursor biomolecules and other value-added products (Qin et al. 2012; Kumar et al. 2020). The biosynthesis of protein in microalgae is closely related with inorganic and organic carbons, and nitrogen metabolisms. Therefore, genetic modification strategies in microalgae have been employed to reprogram the assimilation and metabolism of carbon and nitrogen towards the biosynthesis of amino acids, thereby enhancing the accumulation of protein in microalgae (Zheng et al. 2025). Genetic modification strategies for the production of microalgal protein can generally be classified into two categories, such as mutagenesis-based approaches (Bleisch et al. 2022) and recombinant genetic engineering (Ahmad Kamal et al. 2024). Mutagenesis generates genetic diversity of strains through random modifications of the genome and enables to produce phenotypes with desirable characteristics, such as enhanced protein biosynthesis, altered carbon partitioning, lowered pigment biosynthesis and improved growth performance (Bleisch et al. 2022). In contrast, recombinant genetic engineering consents targeted modification of specific genes in DNA and influences metabolic pathways involved in protein biosynthesis in microalgae (Ahmad Kamal et al. 2024). Both approaches have proved the significant potentiality for improving protein productivity and industrial applications of microalgal strains.

Genetic modification was applied to microalgal biotechnology for the higher yield of proteins from microalgae and commercial production of recombinant proteins having unique biological aspects (Banerjee and Ward 2022). Higher yield of proteins by mutagenesis and the production of recombinant proteins with high biological importances via nuclear or chloroplast transformation in microalgae and higher growth rate aligns with the line of principles of process intensification (Jareonsin and Pumas 2021). Scalable cultivation of microalgae is possible in closed photobioreactors having different architectures according to the requirement of unique situations. The use of a phototrophic growth system of microalgae eliminates or significantly reduces the need for expensive organic carbon sources, thereby lowering the cost of raw materials compared to heterotrophic growth systems (Novoveská et al. 2023). Mutagenesis of wild-type of microalgal strain collectively improves the space-time yield of protein through the optimization of intracellular carbon (photosynthetically carbon or organic carbon) partitioning, nitrogen assimilations and enhanced precursor availability for the protein biosynthesis (Bleisch et al. 2022). Recombinant proteins with therapeutic importance, such as vaccines and monoclonal antibodies command substantially higher market prices than conventional microalgal products, such as pigments or nutritional supplements (Ahmad Kamal et al. 2024). Therefore, a pragmatic shift from bulk low-value biochemicals to high-value biopharmaceuticals through the production of recombinant proteins with unique therapeutic importance significantly improves economic feasibility and return on investment (Zheng et al. 2025). Furthermore, genetic modification in microalgae offers the optimization of metabolic pathways, reducing by-product formation and modulating carbon flux towards target protein biosynthesis (Fajardo et al. 2020). Therefore, protein production by genetically modified microalgae exemplifies the fundamental aspects of process intensification.

#### Mutants of algae for protein biosynthesis

Mutagenesis has been frequently employed in wild-type microalgal strains to improve the protein productivity and biomass yield. Unlike recombinant approaches, which encompass the targeted introduction or modification of specific genes in DNA, mutagenesis creates genetic variations through random alterations in the genome using chemical and physical mutagens (Trovão et al. 2022). Ethyl methanesulfonate (EMS) (Schüler et al. 2020; Jin et al. 2021) is frequently used as chemical mutagens for microalgae. Furthermore, ultraviolet radiation (Ramazanov and Ramazanov 2006) and heavy-ion irradiation (Song et al. 2018) are frequently used as physical mutagens for microalgae. The mutagenic strategy produces genetic variants that could subsequently be screened for the desirable phenotypes able to produce higher amounts of protein than their native form. In microalgae, photosynthetically fixed carbon (autotrophic growth) or organic carbon (heterotrophic and mixotrophic growth) are directed toward the biosynthesis of carbohydrates, amino acids/ proteins, lipids, pigments and other metabolites. The upregulation of protein biosynthesis in mutagenic microalgae is primarily associated with modifications in central carbon metabolism and intracellular carbon partitioning, nitrogen assimilation and amino acid biosynthesis. Pigment- and starch-deficient mutants redirect a greater fraction of assimilated carbon toward amino acid and protein biosynthesis unlikely the biosynthesis of pigment and carbohydrate. Mutation suppresses competing metabolic pathways, particularly the biosynthesis of pigments, starch and lipid, and can redirect carbon flux toward the biosynthesis of amino acid and protein. The carbon metabolic flux reorganization is often accompanied by the upregulation of glycolysis, the tricarboxylic acid (TCA) cycle and amino acid biosynthetic pathways, thereby enhancing the availability of precursors of protein biosynthesis. Furthermore, mutation affects photosynthetic efficiency, light harvesting, nutrient uptake systems and nitrogen assimilation pathways, thereby can significantly influence the biosynthesis of protein in microalgae. Mutation enhances the intracellular pool of nitrogen-containing compounds available for amino acid formation by nitrogen uptake and assimilation influenced by nitrate reductase, nitrite reductase, glutamine synthetase and glutamate synthase. In addition, pigment-deficient mutants may display unique photosynthetic activity which may increase the protein yield, thereby gaining attention in industrial cultivation conditions (Bleisch et al. 2022). However, mutagenesis is less precise than modern genetic engineering techniques to modify wild-type of strain and improve protein productivity, it is considered as an attractive strategy because it does not inevitably involve the introduction of a foreign DNA and thereby, often associated with fewer regulatory constrictions (Trovão et al. 2022).

Chemical-induced random mutagenesis has been explored to get desired phenotype for higher yield of proteins and lower content of pigments in cells. Two mutants of *Chlorella vulgaris* with white (MT02, 99% decrease in chlorophyll content than wild-type) and yellow (MT01, 80% decrease in chlorophyll content than wild-type) color were successfully developed using this strategy. Wild-type *Chlorella vulgaris* was treated with 400 mM ethyl methane sulfonate (EMS) for 1 h under mild agitation in the dark condition followed by the termination of reaction using sodium thiosulfate to a final concentration of 5%. The yellow mutant was subjected to a second round of random mutagenesis using 300 mM EMS and 10 μm norflurazon (carotenoid biosynthesis inhibitor) and incubated at temperature 30 °C in the dark condition for 1 week to get MT02 mutant. Heterotrophic growth of strains was performed in 50 mL culture medium using the concentration of glucose 20 g∙L^−1^ in medium with initial pH 6.5, temperature 30 °C, agitation rate 200 rpm and photon flux density of 100 µmol∙m^−2^∙s^−1^. Concurrently, MT02 exhibited a significantly lower biomass concentration when cultivated in dark condition compared to the wild-type and MT01, and was not able to grow under light conditions. MT02 contained a higher protein content (48.7% in dry basis) than wild-type strain (39.5% in dry basis). The protein content in MT02 was increased 60% compared to wild-type strain; whereas, it exhibited 30% of increase in MT01 compared to the wild-type strain (Schüler et al. 2020). In another investigation, wild-type of *Chlorella sorokiniana* GT-1 obtained from Microalgae Biotechnology Center, State Development and Investment Cooperation, China was considered to develop a starchless mutant SLM2 through chemical mutagenesis (Wu et al. 2019) and in later experiments, mutant was used for the production of protein (Jin et al. 2021). Wild-type of *Chlorella sorokiniana* cells were treated with 0.5% (w/v) EMS (considered as median lethal concentration) for 4 h. After the mentioned treatment, over 35,000 colonies were grown on the modified Endo agar plates (a medium having glucose and inorganic salts without nitrogen sources). Subsequently, colonies were cultivated in the modified Endo growth medium and were exposed to iodine vapor for screening the starchless mutants after growing for 5 days. A colony which was not stained was considered starchless and designated as SLM2. Isolated *Chlorella sorokiniana* GT-1 and its low‐starch mutant SLM2 were cultured separately for 6 days in the modified Endo medium, pH 6 in 7.5 L laboratory fermenter for the protein production. The concentration of glucose and nitrogen source, C/N ratio, incubation temperature in the modified Endo medium were varied. The obtained optimum conditions were nitrogen source potassium nitrate, C/N ratio 16 and the concentration of glucose 30%. Under optimum conditions, protein concentrations were increased from 21.9 to 27.6% of DCW and 27.6 to 37.7% of DCW from initial day to day 6 for wild-type of strain and mutagenic strain, respectively (Jin et al. 2021).

Physical treatment, such as heavy-ion irradiation (HII) has been employed to get the desired phenotype of microalgae for the higher yield of protein and lower content of pigment. Heavy-ion irradiation (HII) mutagenesis was employed to create a mutant of *Chlorella pyrenoidosa* FACHB-9 for the production of protein. In the experiment, the energy of carbon ion was 80 MeV/µ with an average linear energy transfer (LET) value at about 31 keV∙µm^-1^. Cells were irradiated by five different doses, such as 40 Gy, 80 Gy, 120 Gy, 160 Gy, and 200 Gy. The yield of protein in mutant K05 was improved 15.1% (1.92 g∙L^−1^, 54.7% of DCW) compared to FACHB-9 (Song et al. 2018). Furthermore, ultraviolet-induced mutagenesis was considered to produce starchless mutant *Chlorella pyrenoidosa* STL-PI from *Chlorella pyrenoidosa* 82T from the Institute of Plant Physiology, Moscow, Russia. Cell pellet of *Chlorella pyrenoidosa* 82T was resuspended in a medium (200 000 cells·mL^−1^), and the suspension was subjected to ultraviolet irradiation having wavelength 254 nm for 30 min. The ultraviolet-treated mutants were grown in nitrogen-free medium with and without acetate followed by the exposure in iodine vapor to isolate starch-less mutants. The protein content of *Chlorella pyrenoidosa* 82T was 25.2% which was increased to 38.0% in *Chlorella pyrenoidosa* STL-PI (Ramazanov and Ramazanov 2006).

#### Recombinant algae for protein biosynthesis

Genetic engineering approaches have been widely adopted for the production of recombinant proteins with biopharmaceutical importances from microalgae. Microalgae-based recombinant proteins include antibodies, hormones, immunotoxins, cytokines, vaccines, enzymes, nutraceuticals, thrombolytic agents and anti-cancer therapeutics (Yan et al. 2016). Recent advances in genome sequencing and the genome database of microalgae could provide the identification and distribution of gene families involved for the biosynthesis of proteins and metabolic regulation. Nevertheless, only a limited number of whole genome sequences of microalgae are currently available (Shi et al. 2021), which restricts a significant and relevant mapping of the genome, responsible for protein biosynthesis. Up to now, amino acid biosynthesis pathways in *Chlorella* sp. (Xiao et al. 2022) and Diatoms (Bromke 2013) were published. To address these limitations, various omics approaches, such as genomics, transcriptomics, methylomics and proteomics to unravel the intricate metabolic pathways and their regulatory mechanisms within microalgal cell and the use of genetic engineering tools for the improvement of microalgal strain could be considered as an emerging approach for the bioprospecting of proteins from microalgae (Sproles et al. 2021). Furthermore, microalgae could be considered as model eukaryotic hosts, especially where specific genes are related to the function of flagella, photosynthesis and photoreception cannot be expressed. In addition, they could grow in bioreactors in controlled manner under both heterotrophic and phototrophic conditions (León-Bañares et al. 2004). Recently, emerging approaches of genetic engineering including the design of expression vectors, the discovery of genetic regulatory elements (marker genes, reporter genes and promoters) and genetic transformation methods along with bioinformatics algorithms have taken into consideration to develop genetically modified microalgae with the aid of obtaining the higher yield of desired proteins (Barati et al. 2021). More than 50 various types of recombinant proteins were successfully produced from microalgae (Hempel and Maier 2016). Less expensive cultivation process, easy genetic modification, easy modification of proteins after translation and significant growing rates similar to Chinese Hamster Ovary (CHO) cells are driving factors for using microalgae as a source of edible and therapeutic proteins (Fajardo et al. 2020; Sproles et al. 2021). Advantageously, recombinant proteins can be expressed through the transformation of either the nuclear genome or the chloroplast genome in microalgae. Nuclear genome activity permits protein secretion and post-translational modifications, such as formation of disulfide bonds and glycosylation, allowing them as competitive alternatives to mammalian systems. In this case, recombination machinery for integration of exogenous DNA is mostly non-homologous, gene silencing is probable, the level of gene expression (gene copy number) is low or intermediate and co-transformation of different markers is high (León-Bañares et al. 2004). On the other hand, chloroplast genome activity corrects the folding of recombinant proteins, formation of disulfide bonds and improved solubility so as to preserve biological activity inside the plastids (Rasala and Mayfield 2015). The algal chloroplast ensures a higher level of stable expression compared to the nuclear genome. In this case, foreign genes can be precisely integrated on specific loci inside the chloroplast genome via homologous recombination. This process is insensitive to gene silencing and positional effects (Wannathong et al. 2016; Stoffels et al. 2017). Advantageously, the level of gene expression is higher by chloroplast transformation than by nuclear transformation. Unfortunately, some post-translational modifications, such as glycosylation are missed by chloroplast genetic modification (Eichler-Stahlberg et al. 2009; Lingg et al. 2012). Considerable efforts were placed to develop molecular tools for producing high-level transgene expression of both the chloroplast and nuclear genomes. Presently, chloroplast of *Chlamydomonas reinhardtii* (Stoffels et al. 2017) and nucleus of diatoms (Davis et al. 2017) can be considered as a new emerging platform to produce therapeutic proteins. However, the biased codon usage is a vital limitation for the expression of heterologous genes in microalgae; some investigators employed it in some microalgae having extremely high GC content, such as *Chlamydomonas* (GC content 61%) for the production of proteins having pharmaceutical importances (Barahimipour et al. 2016; Wannathong et al. 2016; Hirschl et al. 2017; Lima et al. 2018; Tevatia et al. 2019; Kiataramgul et al. 2020; Wang et al. 2020). Expression of an exogenous gene in microalgae could be non-existent or occur at very low level; however, all the elements for optimum transcription and translation can be achieved by incorporating essential regulatory elements, such as promoters, introns, and other regulatory regions, into the chimeric gene construct. Furthermore, if transgenic algal clones are not maintained under selected conditions, expression of the exogenous gene might be suppressed or lost (León-Bañares et al. 2004). The gene silencing is attributed to a variety of epigenetic mechanisms. Silencing of transgenes remains the prime limiting issue for the stable expression of foreign genes; however, this problem is not unique for microalgae. Recently, several elements involved in the silencing of transgenes in *Chlamydomonas* sp. have been identified (Wu-Scharf et al. 2000). Strategies for producing different recombinant proteins from microalgae having biopharmaceutical potential are presented in Table 1.

**Table 1:** Genetic engineering of microalgae for protein production

Microalgae	Gene/ target site	Approach	Outcomes	References
*Chlamydomonas reinhardtii*	Endolysins Cpl-1 and Pal	Foreign gene expression	Recombinant protein yield: ~1.3 mg/g algal dry weight	Stoffels et al. (2017)
*Chlamydomonas reinhardtii*	Birch pollen allergen Bet v1	Codon-optimized gene and stably integrated	Allergen expression with yields between 0.01 and 0.04% of TSP	Hirschl et al. (2017)
*Chlamydomonas reinhardtii*	HIV antigen P24	Codon-optimized	Yield of recombinant protein increased up to 0.25% of the total cellular protein	Barahimipour et al. (2016)
*Chlamydomonas reinhardtii*	Human interferon (IFN)	Cloning and expression	IFN-α2a was expressed and it had anticancer and antiviral activities	El-Ayouty et al. (2019)
*Chlamydomonas reinhardtii*	PfCelTOS Antigen	Chloroplast expressed	Expressed recombinant PfCelTOS accumulates as a soluble protein	Shamriz and Ofoghi (2019)
*Chlamydomonas reinhardtii*	Human growth hormone (hGH)	Codon-optimized and new vectors	0.5 mg hGH per liter of culture	Wannathong et al. (2016)
*Chlamydomonas reinhardtii*	*cdo* and *csad* genes	Codon optimized and transformed as either separate genes (*cdo*::*csad*) or fusion protein (*cdo*-*csad*)	Improve taurine synthesis of pathway intermediates by about 7-fold	Tevatia et al. (2019)
*Chlamydomonas reinhardtii*	Nucleotide encoding a C-terminal hemagglutinin (HA) and 6×His double tagged 3×ToAMP4 with an EcoRI and an XhoI site located at its 5′ and 3′ end, respectively	Transformation to *C. reinhardtii* nuclear expression vector pBSK-RBCS2-Ble-FMDV2A^pro^	Antibacterial activity (gram-positive and gram-negative bacteria), low hemolysis activity and low cytotoxicity	Xue et al. (2020)
*Chlamydomonas reinhardtii*	hVEGF-165 transgene expression	Combination of the *Chlamydomonas reinhardtii* UVM11 with the pBC1 vector	Endothelial cell tube formation ability increased by human pro-angiogenic growth factors	Jarquín-Cordero et al. (2020)
*Chlamydomonas reinhardtii*	Intrinsic factor expression cassettes	Codon-optimization	Human intrinsic factor protein by Enriched vitamin B12 in cell lysates	Lima et al. (2018)
*Chlamydomonas reinhardtii*	WSSV VP28 gene	Codon-optimization	Improved survival rate of infected shrimp by white spot syndrome virus protein	Kiataramgul et al. (2020)
*Chlorella* sp.	Plasmid pMDC85-Scy/hepc DNA	Scy-hepc fusion gene was transformed by electroporation.	Marine antimicrobial peptides and increased survival of fish	He et al. (2018)
*Chlamydomonas reinhardtii* and *Chlorella vulgaris*	SARS-CoV-2 rEceptor binding domain (RBD) and basic fibroblast growth factor (bFGF)	Nuclear transformation	1.14 mg/g RBD and 1.61 ng/g FGF in *Chlorella vulgaris.* 1.61 mg/g RBD and 1.025 ng/g FGF in *Chlamydomonas reinhardtii*.	Malla et al. (2021)
*Schizochytrium sp.*	Epitopes from tumor associated antigens	Cloning and expression	BCB protein was expressed at levels up to 637 µg/g fresh weight.	Hernández-Ramírez et al. (2020)
*Schizochytrium sp.*	LTB: RAGE vaccine	Algevir system (inducible geminiviral vector)	Increase the yield up to 380 µg LTB: RAGE/g fresh weight.	Ortega-Berlanga et al. (2018)
*Schizochytrium sp.*	Vaccine against Zika virus (ZIKV)	Algevir technology to express an antigenic protein	Antigen yields of up to 365 µg/g microalgae fresh weight.	Márquez-Escobar et al. (2018)
*Haematococcus pluvialis*	Antimicrobial peptide piscidin-4	Expression of codon-optimized	Confirmed that the antimicrobial peptide could be expressed from *Haematococcus pluvialis.*	Wang et al. (2020)
*Thalassiosira pseudonana*	Antigen IbpA DR2	Nuclear-based expression	Increased recombinant protein production by 1.2%	Davis et al. (2017)
*Tetraselmis subcordiformis*	*rt*-PA	Nuclear transformation	*rt*-PA was integrated	Wu et al. (2021)
*Fistulifera solaris*	*cox* gene	Cloning and expression	The total content of Prostaglandins (PGs) was 1290.4 ng/g dry cell weight	Maeda et al. (2021)

The metabolic pathways underlying enhanced protein biosynthesis in genetically modified microalgae is primarily connected with the modulation of intracellular carbon and nitrogen metabolisms. Genetic modification redirects metabolic fluxes from competing storage pathways (the biosynthesis of starch and lipid) to the biosynthesis of amino acids and protein formation in microalgae. Furthermore, genetic modifications in microalgae enable optimization of metabolic pathways, reducing the formation of by-products and redirecting carbon flux toward the biosynthesis of targeted protein (Chen et al. 2025). This metabolic redirection is associated with the overexpression of key enzymes involved in carbon fixation and amino acid biosynthesis pathways. Furthermore, enhanced expression of nitrogen assimilation genes, including nitrate and ammonium transporters, and enzymes, such as glutamine synthetase (GS), glutamate synthase (GOGAT), glutamate dehydrogenase (GDH), asparagine synthetase (ASN), nitrate reductase (NR) and nitrite reductase (NiR) improve the intracellular supply of nitrogenous items as protein precursors. Furthermore, photosynthetic efficiency in engineered microalgae increases the availability of ATP and NADPH, which are indispensable energy carriers for the biosynthesis of protein. Collectively, these metabolic adjustments in recombinant microalgae result in higher precursor availability for the formation of intracellular protein (Sproles et al. 2021). Furthermore, microalgae have the ability to perform post-translational modifications, which are essential for the many recombinant proteins having unique therapeutic importance, allowing them as competitive alternatives to mammalian systems (Ahmad et al. 2020).

However, it has been reported that the acceptance of both microalgae and macroalgae as food has been documented among indigenous tribes in Chile dating back nearly 14,000 years and across several Asian countries over centuries; the modern and systematic utilization of microalgae began only in the 1950s, primarily as a food supplement (Rajauria and Yuan 2021). Since then, regulatory recognitions have played a pivotal role in evolving microalgae-based food formulations. Several microalgal species have been listed in the EU Novel food catalogue for food applications by the European Food Safety Authority (EFSA) and approved by the United States Food Drug and Administration (FDA) (Ferreira de Oliveira and Bragotto 2022). There are 200,000-800,000 microalgae species present in nature to date (Koyande et al. 2019); however, only less than 10 are produced commercially (Krishna Koyande et al. 2020). In last decade, microalgae, such as *Arthrospira platensis*,* Auxenochlorella protothecoides*,* Chlamydomonas reinhardtii*,* Chlorella vulgaris*,* Dunaliella bardawil* and *Euglena gracilis* gained enormous attentions for the formulations of foods and biopharmaceuticals because of their long history of consumption and they offer wide ranges of bioactive compounds (Chacón-Lee and González-Mariño 2010; Torres-Tiji et al. 2020).

Approximately 20 different microalgal species including cyanobacteria have been successfully genetically modified. Among them, *Chlamydomonas reinhardtii* was given special attention in several investigations (Charrier et al. 2015). In spite of the considerable successful achievement in the genetic engineering of microalgae, legal frameworks have been consistently considered as a bottleneck for microalgae stakeholders, regardless of their added-value metabolites and nutritional values (Janssen et al. 2022; Cruz and Vasconcelos 2024). Regulatory frameworks and legal approval related to implementation of genetically modified organisms including microalgae in food and feed formulations vary substantially among countries around the globe, creating uncertainties for the large-scale production and commercialization of genetically engineered microalgae and their metabolites (Onn et al. 2025). Genetically modified microalgae intended for food and feed applications are subjected under rigorous risk assessments established under genetically modified organism legislations in the European Union and the United State of America (Stilgoe et al. 2013). These assessments typically assess potential impacts of genetically modified microalgae on human and animal health, allergenicity, toxicity, environmental safety and ecological persistence. Therefore, the approval process to consider genetically modified microalgae in food and feed formulations can be scientifically demanding, time consuming and expensive, thereby may dampen industrial investment and production of genetically modified microalgae-based products (Wesseler et al. 2023).

The primary regulatory concern is the exposure of genetically modified microalgae into ecosystems. Unlike closed-system photobioreactor, microalgae are frequently cultivated in open-system photobioreactor (outdoor pond) or large-scale photobioreactors where accidental discharge cannot be completely left out. Therefore, regulatory authorities are concerned about the detailed assessments of survivability, competitiveness and the ecological behavior of genetically modified microalgae within the wide ranges of environmental conditions (Kumar et al. 2020). Particular attention has been placed to comprehend the possibility of horizontal gene transfer among genetically modified microalgae and native microbial communities, unintentional ecological interactions and the modulation of native microbial communities (Levine et al. 2004).

The regulatory assessment for the genetically modified microalgae is additionally complicated by the diversity of genetic engineering techniques currently accessible. Modern genome-editing techniques, such as CRISPR/Cas systems offer targeted modifications in DNA without introducing exogenous genetic material; whereas, traditional transgenic techniques often consider the introduction of foreign DNA sequences, originating from unrelated organisms (Baek et al. 2016; Ferenczi et al. 2017). In several authorities, uncertainty remains regarding whether genome-edited microalgae could be measured in the similar way as conventional transgenic organisms. This difference has become increasingly significant because genome editing offers opportunities to develop microalgae strains with enhanced protein productivity while minimizing genetic modulations and potential biosafety aspects (Szyjka et al. 2017).

In addition to regulatory approval, public discernment epitomizes another important aspect for the commercialization of genetically modified microalgae. There is no information about commercially-available food products produced by genetically modified microalgae until now. Concerns about food safety and transparency of labeling may influence market adoption of microalgae-derived proteins produced using genetic engineering technologies (Bassani et al. 2025). Consequently, effective communication of scientific evidence, transparent risk assessment procedures and clear regulatory frameworks related to genetically modified microalgal products are prerequisite for improving consumer acceptance (Spicer and Molnar 2018). Consumer acceptance of microalgae-based foods remains variable across different geographical regions in the globe. According to a consumer survey with 940 consumers from three different European countries (Netherlands: 310, Germany: 315 and France: 315), it was found that consumers have preference and willingness to consume microalgae-based products as a substitute for meat products (Onwezen et al. 2021).

### Bioprocess aspects

The upstream bioprocess technology for the cultivation of microalgae is an essential step for the overall process because it directly affects the yield of protein and subsequent downstream technology. The synthesis of protein in the biotic phase of microalgae is strongly linked to environmental and nutrient conditions, such as (a) the intensity of sunlight and photoperiod, (b) level of CO_2_ or bioavailable carbon in environment, (c) pH of growth medium, (d) atmospheric temperature, (e) available nutrients (nitrogen, phosphorus and potassium) and (f) the presence (or absence) of other organisms (Juneja et al. 2013). Furthermore, the type and species of microalgae significantly influence the biosynthesis of protein as different taxa exhibit distinct metabolic capacities and nutrient assimilation efficiencies based on their cultivation systems (Uma et al. 2022). Operational parameters of the bioreactor are directly linked with operational strategies of the bioprocess, thereby play significant roles in directing metabolic fluxes toward protein biosynthesis in microalgae.

#### Decisive operational parameters of bioreactor

The effective implementation of process intensification strategies for the production of proteins from microalgae fundamentally depends on the understanding of individual operational parameters and their physiological impacts in an inclusive way. Microalgae are highly sensitive to culture conditions including light intensity, light quality, photoperiod, nutrient availability, temperature, pH and nutrient stress because these directly influence their metabolic pathways. In particular, protein biosynthesis in microalgae is tightly regulated by photosynthetic efficiency, carbon and nitrogen bioavailability, and cellular energy balance. Without a comprehensive understanding of how each operational parameter independently and synergistically affects cellular metabolism, alternation of process parameters may result in suboptimal yield of proteins or unintentional metabolic shifts towards the synthesis of other biomolecules, such as lipids and carbohydrates. For example, increasing light intensity to improve photosynthesis may consequently induce oxidative stress or photoinhibition, thereby reducing cellular viability and protein yield. Furthermore, nutrient limitation strategies designed to manipulate the composition of algal biomass. Therefore, protein productivity could be modulated if nutrient availability is not precisely controlled. The interactions among different operational parameters are often non-linear, signifying that optimizing one operational parameter without considering others may not offer improved yield of proteins from microalgae. Understanding the physiological responses by altering operational parameters enables the rational cultivation strategies of microalgae tailored specifically for maximizing protein productivity by microalgae. Combining microalgal physiological responses by operational parameters with engineering design principles ensures the process intensification strategies are scientifically grounded rather than empirically driven. The effects of the light spectrum, intensity and photoperiod, temperature, and the concentrations of carbon (organic and inorganic) and nitrogen on the protein content in algal cells vary among species. Therefore, the understanding of species-specific responses of aforementioned factors is an imperative aspect for the utmost rate of algal protein production (Chen et al. 2025).

*Light spectrum*,* intensity and photoperiod*

Light regime is one of the most decisive operational parameters that influence the metabolic regulation, protein biosynthesis and biomass productivity of microalgae under photoautotrophic, photoheterotrophic and mixotrophic cultivation strategies. Each cultivation strategy exhibits unique metabolic flux distributions, redox balances and energy generation biochemical pathways, resulting in species-specific and condition-dependent variations in protein biosynthesis. The protein biosynthesis in microalgae is significantly impacted by the interplay between light spectrum, intensity and photoperiod (Lehmuskero et al. 2018). In photoautotrophic cultivation strategy, light acts as the sole energy source of photosynthetic electron transport and inorganic carbon (CO_2_) assimilation, thereby directly controls the cellular energy availability (ATP and NADPH generation) required for amino acid biosynthesis (Rivera-Sánchez et al. 2025). Unlike photoautotrophic cultivation strategy, heterotrophic cultivation strategy is not dependent on light energy, as microalgae rely entirely on organic carbon sources for energy generation and biomass formation (Xu et al. 2021; Jin et al. 2021; Xiao et al. 2022). Photoheterotrophic growth characteristics of microalgae represents a combined strategy, integrating light-driven photosynthesis along with assimilation of organic carbon. In this strategy, light intensity and photoperiod interact synergistically with organic carbon metabolism to increase cellular energy supply and in later extent metabolic flexibility (Wang et al. 2014a). In mixotrophic cultivation strategy, protein biosynthesis by microalgae is strongly influenced by the combined effects of light spectrum, intensity and photoperiod, which together coordinate photosynthesis and respiratory metabolism in microalgae (Matos et al. 2017; Abiusi et al. 2022; de Mendonça et al. 2022). Light intensity and wavelength control the extent of photosynthetic electron transportation and increase the cellular energy supply. This energy accompanies respiratory metabolism derived from organic carbon assimilation, thereby improving the overall energy supply and increasing the availability of reducing power for amino acid biosynthesis in microalgae (Wang et al. 2014b).

**Fig. 2:**
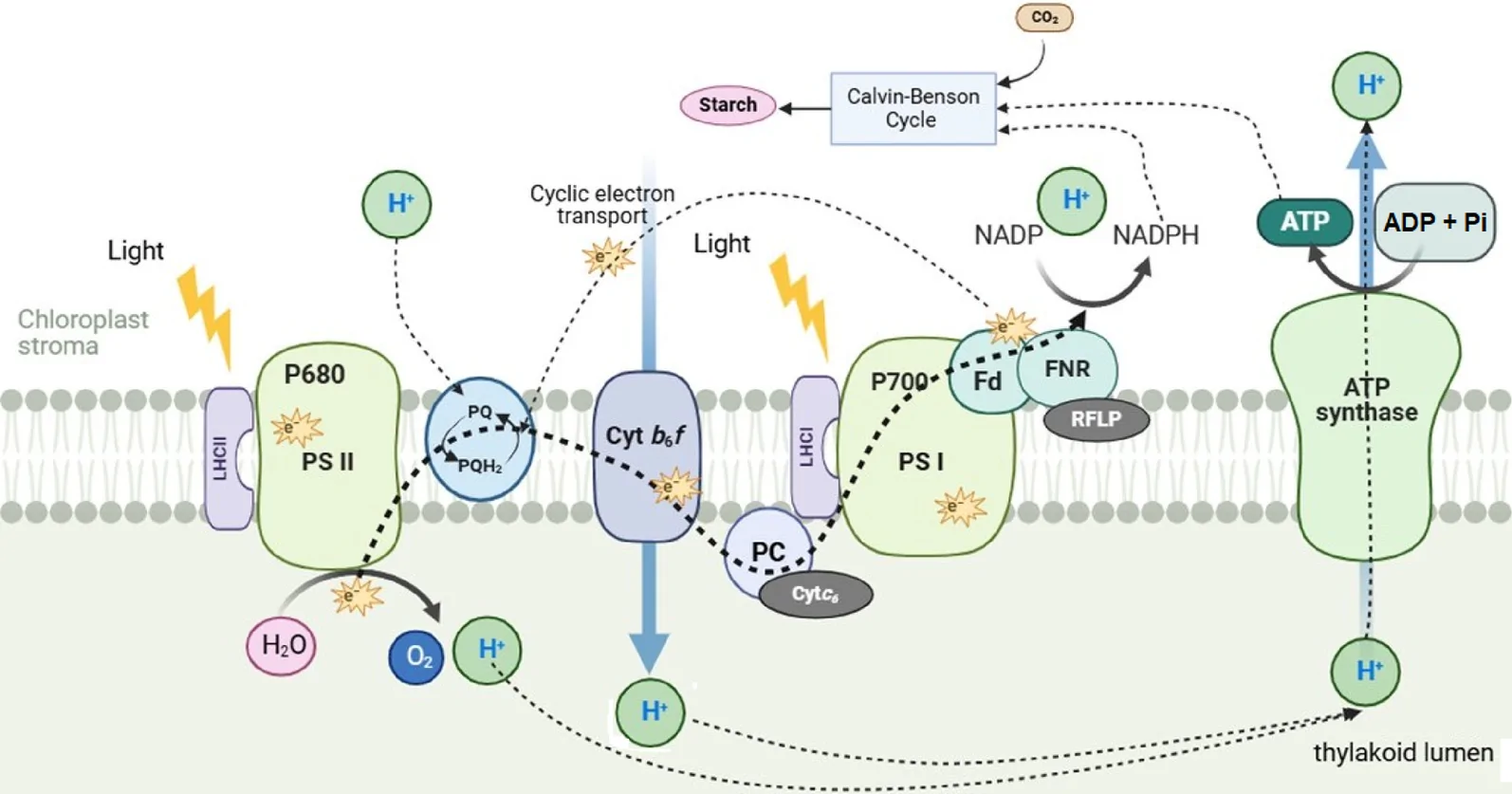
Biochemical involvement of photosystems (PSI and PSII) for the absorption of light and photosysnthesis in microalgae for CO_2_ assimilation. Photosystem II (PSII), located in the thylakoid membrane of the chloroplast, absorbs photons to catalyze the photolysis of water, producing electrons, protons, and molecular oxygen, thereby initiating the light-dependent electron transport chain. Electrons are transferred sequentially through the plastoquinone pool, cytochrome b₆f complex and plastocyanin to Photosystem I (PSI), also localized in the thylakoid membrane, where they are further energized by light absorption. PSI facilitates the reduction of NADP⁺ to NADPH, while the concomitant establishment of a proton motive force across the thylakoid membrane drives ATP synthesis via ATP synthase. These light-dependent reactions provide the chemical energy (in the form of ATP) and reducing power (in the form of NADPH) required for the Calvin-Benson cycle, the light-independent (dark) phase occurring in the chloroplast stroma, which assimilates CO₂ into triose phosphates (C3 pathway) and ultimately carbohydrate (glucose unit). Collectively, PSII and PSI orchestrate the coupling of photochemical energy conversion with carbon fixation, thereby sustaining photosynthetic productivity in microalgae (self-developed, concept was adopted from Grechanik and Tsygankov (2022)

The primary step of the solar-driven metabolic pathway in microalgae is light absorption and transformation to biochemical energy (ATP and NADPH). Similar to terrestrial plants, thylakoid membranes of chloroplast in microalgae contain specialized photosystems (PSs), such as PSI and PSII. Both photosystems absorb light energy to execute photosynthesis. PSII absorbs photons from sunlight and produces two electrons by the photolysis of water (the oxidation of water at the oxygen-evolving complex (OEC) of PSII). Electrons are transferred from PSII by plastoquinone (PQ) which is converted to plastoquinol (PQH_2_) and subsequently, the cytochrome b6f complex (Cytb6f). The transfer of electrons from Cytb6f to PSI is mediated by either plastocyanin (Pc) or cytochrome c_6_ (Cytc_6_). Furthermore, PSI can be excited by light wavelength and translocates electrons to Ferredoxin-NADP+ reductase (FNR) in PSI. Electrons are accepted by FNR and produce NADPH. ATP produced by ATP synthase and NADPH in the light phase biochemical reactions are utilized in the Calvin-Benson-Bassham cycle, shortly known as Calvin cycle where starch is produced from carbonaceous molecules. Derivatives of starch is subsequently metabolized through glycolysis and the TCA cycle, supplying precursor molecules, such as α-ketoglutarate and oxaloacetate for the biosynthesis of amino acids (Yang et al. 2000; Qian et al. 2017). Therefore, light-harvesting into the photosynthetic electron transport chain is an important aspect to balance energy supply with metabolic demands and to suppress the formation of dangerous reactive oxygen species (ROSs). The LHC proteins play an important role for fine-tuning the activity of PSII within a short time period (Milrad et al. 2024). It may be realized that light wavelength and intensity determine the rate of the light reactions in photosystems I and II in the thylakoid membrane. Insufficient light supply restricts the activity of these energy carriers; whereas, excessive light supply may cause oxidative stress and photoinhibition, resulting in dysregulated biosynthetic processes (Mussgnug et al. 2007; Hu et al. 2023). Furthermore, photosynthetically-derived reducing power enhances nitrogen assimilation through the upregulation of nitrate reductase and the glutamine synthetase-glutamate synthase (GS-GOGAT) pathway, linking light accessibility directly to the biosynthesis of amino acids (Tischner and Hüttermann 1980; Weger et al. 1988). Furthermore, light quality (spectrum) modulates photosystem excitation in a unique way, thereby influencing carbon and nitrogen assimilation pathways, and ultimately affecting biomass productivity and protein yield. Red and blue wavelengths are particularly relevant in case of protein biosynthesis in several microalgal species because they could modulate the expressions of genes involved in carbon transport, nitrogen assimilation and protein translation in light-dependent growth of microalgae. It has been proven that blue light is associated with enhanced nitrogen uptake and regulatory signaling; whereas, red light is often linked to higher biomass productivity and protein biosynthesis in several green microalgal species (Borodin 2008; Esteves et al. 2025). The photoperiod further modulates the duration of CO_2_ assimilation in microalgae through circadian-regulated metabolic pathways by controlling light-dark cycle. This light-dark cycle regulates transcriptional activity and the turnover of photosynthesis-associated proteins, such that extended or continuous illumination may enhance the yield of protein from microalgae but also increases the risk of photooxidative stress leading to photodamage of LHC proteins of photosystems (Redekop et al. 2022). Therefore, optimization of light spectrum, light intensity and photoperiods in light-driven photosynthesis process can reduce photoinhibition while sustaining higher yield of protein, making the strategy particularly attractive for high-value protein production from microalgae. Biochemical involvement of photosystems in microalgae for the absorption of light and photosynthesis is presented in Fig. 2.

For a long time, open ponds are used in commercial-scale production of microalgae due to their low construction costs and non-complicated operations. Natural and artificial water systems can be used as open systems for microalgal growth. Natural open systems are lakes, ponds and lagoons. Artificial open cultivation systems are man-made tanks, ponds and containers with a variety of sizes and a greater degree of controlling system and accessories (Xu et al. 2009; Singh and Sharma 2012). One of the limiting factors in cultivating microalgae in outdoor open pond cultivation systems is their photoinhibition caused by high intensity of light and lower penetration of sunlight into the bottom of the cultivation system. The lower-light intensity increases the transcription of the LHC proteins to maximize photon capture efficiency; whereas, when light intensity exceeds energy demand by cells, the transcription of LHC proteins is down-regulated to prevent photodamage. In order to reduce this problem, changing the expression of key genes in both steps of the photosynthetic pathway (light phase and dark phase), and non-coding RNAs (ncRNAs) and transcription factors (TFs) associated with the photosynthetic pathway can effectively improve the biomass productivity and the yield of protein from microalgae (Hu et al. 2023). RNA interference (RNAi) technology was employed to reduce the risk of oxidative photodamage by downregulating the expression of the LHC proteins associated with photosystems I and II (PSI and PSII), such as LHCI and LHCII in wild-type of *Chlamydomonas reinhardtii*. Compared to wild-type of *Chlamydomonas reinhardtii*, ⁓30% improvement in photosynthetic efficiency was noted in genetically altered algal strain Stm3LR3. The photoinhibition could occur in cells nearest the wall of the reactor by high light intensity and up to 90% of the light energy could be wasted. Therefore, cells inside of the bioreactor could receive lower amounts of light energy not only from this wasted energy, but also from shading occurring by cell-layer on the surface of the bioreactor. By decreasing the sensitivity of PSII to high light intensity, less amount of light energy could be wasted and a better light penetration into the bioreactor could be possible. It may be realized that it could be possible to increase the bioreactor volume with this strategy (reduced risk of oxidative photodamage of PSII in microalgae) (Mussgnug et al. 2007).

Optimization of light illumination conditions is considered as an important strategy for enhancing protein productivity from microalgae. Therefore, appropriate selection and adjustment of these light parameters for each strain can significantly increase biomass production and protein yield during the cultivation of microalgae. However, light wavelength, intensity and photoperiod are strongly related to photosynthetic efficiency, cellular metabolism and ultimately protein biosynthesis in microalgae, their effects vary among species. Several researches focused on the effects of light wavelength, light intensity and photoperiod on the growth of microalgae and protein biosynthesis to identify optimal illumination conditions for enhanced protein yield.

It was proven that the blue light improves the biosynthesis of protein in *Spirulina platensis* compared to other light sources (yellow, red, green and white) and it increased with time progress. Protein yield was improved with increasing green-light intensity 9–54 µmol photon·m^−2^·s^−1^; however, it was reduced by increasing light intensities of white and yellow light sources. Interestingly, photo-inhibition was not linear with increasing light intensities of red and blue light sources (Zhang et al. 2024). In the case of *Chlamydomonas reinhardtii*, the combined conditions of blue light (constant led light intensity 105 µmol·m^−2^·s^−1^) and a temperature of 28–24 °C improved the protein content than white-yellow light at temperature of 32 °C. The protein content was decreased at temperature of 32 °C for all types of lights (blue, red-orange and white-yellow) (Li et al. 2021). Other investigation demonstrated that blue or green light with a higher proportion of white light could increase the protein content in *Spirulina* sp.; however, the improvement of protein content was not statistically significant compared to blue light (Prates et al. 2018). Contradictorily, it has been reported that the highest protein productivity of *Spirulina* sp. could be obtained by red and green light sources having intensity 500 µmol photon·m^−2^·s^−1^, and together with white light source having intensity 41.6 µmol photon·m^−2^·s^−1^ (da Fontoura Prates et al. 2020). Some investigations indicated that red and white light sources could improve the synthesis of protein in *Tetraselmis suecica* but it is not statistically significant compared to blue and green light when constant light intensity 160 µmol photon·m^−2^·s^−1^ was used (Abiusi et al. 2014). Effects of light wavelength along with light cycle were investigated for several strains of microalgae with the aid of higher protein yield. During cultivation of *Euglena anabaena*, constant light/dark cycle was maintained 24:0 for 16 days; whereas, light wavelength was varied, such red-light (680 nm) and blue-light (470 nm) considering white-light as a control and constant light intensity 60 µmol m^−2^ s^−1^. It was found that white-light promoted higher cell density; whereas, red-light promoted higher biomass growth. The protein content was higher by white light wavelength compared to red light wavelength; however, results were not significantly different (Wulandari et al. 2025). Concentration of protein in *Dunaliella salina* was significantly increased by blue and red-light treatment (second stage of cultivation process) after white light treatment (first stage of cultivation process). The protein productivity was improved by 200 and 400 µmol photon·m^−2^·s^−1^ of both blue and red-light intensities compared to 100 µmol photon·m^− 2^·s^− 1^ of light intensity; however, 600 µmol photon·m^− 2^·s^− 1^ of blue-light intensity may cause inhibition (Sui and Harvey 2021). Furthermore, it was found that biosynthesis of protein was higher in *Dunaliella salina* by fractional photoperiods (12 h : 12 h light : dark) with light intensity 55 µmol·m^−2^·s^−1^ compared to continuous photoperiod (Sui et al. 2019). Contradictorily, continuous light photoperiod (24 h : 0 h light : dark) with intensity 150 µmol·m^−2^·s^−1^ resulted in higher biosynthesis of protein (47.3%) compared to fractional photoperiods (12 h : 12 h light : dark) in *Scenedesmus obliquus* (Vendruscolo et al. 2019). Concentration of protein in *Chlorella vulgaris* was maximum (46%) by light intensity 100 µmol photon·m^−2^·s^−1^ and fractional photoperiods (16 h : 8 h light : dark); whereas, protein concentration was minimum (33%) by light intensity 37.5 µmol photon·m^−2^·s^−1^ and opposite way of light-dark fractional photoperiods (Seyfabadi et al. 2011). *Chlorella minutissima* exhibited enhanced protein productivity under lower light intensity (8.44 µmol photon·m^−2^·s^−1^) and fractional photoperiods (12 h : 12 h light : dark) when it was cultivated in a medium having pentose sugar (d-xylose and/or l-arabinose). Photoinhibition of *Chlorella minutissima* was noted by light intensity 33.75 µmol photon·m^−2^·s^−1^ in all cultivation medium (Freitas et al. 2017). However; photoinhibition was noted for *Chlorella minutissima*, the biosynthesis of protein was improved by increasing light intensities (100–300 µmol photon·m^−2^·s^−1^) in three unnamed mixed microalgae from three different water-bodies with ecologically diverse functional properties, nearby Kasumigaura Lakeside and also Bio-eco Engineering Laboratory of National Institute of Environmental Studies in Tsukuba, Japan (Kumar et al. 2019). Likewise, when protein production was performed in a bubble column photobioreactor operated with a semi-continuous mode by *Micractinium pusillum* and a total 24 days experiment consisted of three cycles, such as a 10-day cycle in the late exponential phase followed by two 7-day cycles in the mid-exponential phase, it remained steady during light treatment ranging from 29.38% to 27.76%, but decreased slightly (35.8–28.9%) with increasing light intensity (50–650 µmol·m^−2^·s^−1^) combined with a light/dark cycle of 24:0 (Singh Chauhan et al. 2023). On the other hand, protein biosynthesis was not changed in *Ankistrodesmus falcatus* when it was cultivated in Bolds Basal Medium (BBM), CHU-10 and Zarrouk’s Medium (ZM) under incubation temperature 25 °C with fixed light intensity of 60 µmol·m^−2^·s^−1^ but different light intensities (30–150 µmol photon·m^−2^·s^−1^) and photoperiods (12 h : 12 h, 18 h : 6 h, 6 h : 18 h, 24 h : 0 h and 0 h : 24 h light : dark) (George et al. 2014).

However; these examples did not adequately address the underlying molecular mechanisms responsible for the practical variations among strains, a comprehensive understanding of how the expression of LHC proteins associated with PSI and PSII responds to different light conditions and subsequently regulates microalgal growth and protein biosynthesis could provide an idea.

*Temperature*

Temperature exerts a substantial impact on the biomass of microalgae and protein yield through its regulatory effects on metabolic pathways and overall cellular physiological functions. More precisely, temperature influences the activities of enzymes that govern metabolic reactions in the biotic phase, thereby affecting the rates of photosynthesis, respiration, the assimilation of nutrients and the biosynthesis of metabolites. It also modulates the solubility and diffusion of CO₂ in the cultivation medium, directly influencing carbon bioavailability for photosynthetic carbon fixation. Optimal temperature enhances cell doubling time, synthesis of chlorophyll and photosynthetic performance, membrane stability and substrate transportation from abiotic phase to biotic phase, enzyme efficiency and protein accumulation. Elevated temperatures often accelerate metabolic processes but may lead to photooxidative stress and protein denaturation, while low temperatures reduce enzymatic activity and overall growth rate of microalgae. Therefore, maintaining an optimal temperature range is critical for maximizing both biomass productivity and protein synthesis in microalgae (Singh and Singh 2015).

From an engineering perspective, maintaining optimal temperature in large-scale bioreactors for the production of proteins from microalgae often requires substantial energy input for heating or cooling. The use of cold-adapted microalgal strains offers a promising aspect for process intensification, because they are capable of maintaining metabolic activity and protein biosynthesis at relatively low temperatures. Cold-adapted microalgae may exhibit efficient enzymatic systems that remain active under low-temperature conditions, allowing stable biomass production and protein biosynthesis in colder climates or during winter season. Consequently, the requirement for heating of cultivation systems can be reduced, which decreases operational energy consumption and improves economic feasibility of large-scale protein production from microalgae. This aspect enables the production of proteins from microalgae throughout the year and enhances overall process productivity with minimal expenses of heating of the cultivation system, which aligns with the concept of process intensification. To evaluate the feasibility of low-temperature cultivation strategies, several researchers have examined the growth and protein production performance of different microalgal species under psychrophilic and ambient conditions. Selected examples from scientific articles about protein productivity of cold-adapted microalgae are discussed below; however, the energy requirements and associated expenses related to heating for bioreactor considered for microalgal growth and protein production are not addressed due to the lack of information in scientific articles.

In a laboratory-based batch-mode cultivation investigation (cultivation in 6 L round, flat-bottom flasks) a U-shaped relationship between protein content and temperature in the range of 10–25 °C was noted for *Thalassiosira pseudonana*,* Phaeodactylum tricornutum* and *Pavlova lutheri*; whereas, a bell-shaped relationship was noted for *Chaetoceros gracilis*. Protein content decreased in *Chaetoceros simplex* and *Isochrysis aff. galbana* at a similar temperature range; whereas, protein content remained slightly increased in *Dunaliella tertiolecta* and *Chaetoceros calcitrans* with the increase in temperature (Thompson et al. 1992). *Chlorella* UMACC 234, *Chlorella* UMACC 237, *Chlamydomonas* UMACC 229, *Navicula* UMACC 231, *Stichococcus* UMACC 238 and *Klebsormidium* UMACC 227 were cultivated in Erlenmeyer flasks at batch mode with different incubation temperatures, such as 4 °C, 6 °C, 9 °C, 14 °C, 20 °C and 30 °C. All species, excluding *Navicula UMACC* 231 and *Stichococcus UMACC* 238 demonstrated higher amounts of proteins when grown at low temperatures, such as 6 °C and 9 °C (Teoh et al. 2004). *Pseudoneochloris marina* was cultivated in the flat-panel airlift photobioreactors filled with 2.16 L of modified f/2 medium for 7 days under different temperature (20 °C, 28 °C and 36 °C) and light intensity (140 µmol·m^−2^·s^−1^, 252 µmol·m^−2^·s^−1^, and 364 µmol·m^−2^·s^−1^). It was found that the biomass productivity was 0.26 g·L⁻^1^·d^−1^ at temperature 28 °C, f/2 medium having 74.1 mg·L⁻^1^ N-NO₃ and 252–364 µmol·m⁻^2^·s^−1^ light intensity. The protein content was highest (236 mg·g⁻^1^) at temperature 20 °C and light intensity 140 µmol·m^−2^·s^−1^ (Gonçalves et al. 2019). Eight cold-adapted microalgae, such as *Chlamydomonas* sp. MALINA (RCC 2488), *Chlamydomonas* sp. CEFAS (RCC 2607), *Chlorella stigmatophora* (RCC 661), *Tetrasemis chuii* Butcher (SAG 1.96), *Tetraselmis* sp. (RCC 2604), *Nannochloropsis granulata* (RCC 2478), *Phaeodactylum tricornutum* (RCC 641) and *Pseudopleurochloris antarctica* (SAG 39.98) were cultivated in a glass bubble-tube photobioreactor, placed in a climate chamber for 14 days. Four growth conditions were tested to assess the microbial protein production, mentioned herein: (a) low temperature and low light (temperature 8 °C and light intensity 50 µmol·s^−1^·m^−2^) (b) low temperature and high light (temperature 8 °C and light intensity 100 µmol·s^−1^·m^−2^); (c) high temperature and low light (temperature 15 °C and light intensity 50 µmol·s^−1^·m^−2^) and (d) high temperature and high light (temperature 15 °C and light intensity 100 µmol·s^−1^·m^−2^). In all cases, air enriched with CO_2_ (1%v/v) at a flow rate of 110 mL min^− 1^ was provided to the culture medium. Among the strains, *Chlamydomonas* sp. MALINA (RCC 2488) had better growth at a temperature of 8 °C compared to 15 °C (0.53 g DW·L^−1^·d^−1^) and highest protein productivity 70 mg·L^−1^·d^−1^ yielding 37.7% of protein. *Nannochloropsis granulata* and *Phaeodactylum tricornutum* contained 41.6% and 37.7% of proteins. Two tested *Tetraselmis* strains, such as SAG 1.96 and RCC 2604 achieved highest biomass productivities (0.7–1 g DW·L^−1^·d^−1^) and contained up to 15% protein. *Pseudopleurochloris antarctica* (SAG 39.98) grew satisfactorily (0.4 g·L^−1^·d^−1^) at temperature 15 °C and contained 23% proteins (Schulze et al. 2019).

Another important aspect of temperature-related process intensification for the production of proteins from microalgae is the selection of strains which can grow in ambient temperature without significant loss of metabolic activity. Such strains can be cultivated in outdoor systems, thereby reducing the need for heating and cooling systems and minimizing operational costs. Therefore, several researches have explored the cultivation of microalgae under outdoor or ambient environmental conditions to understand the biomass production of microalgae and protein accumulation. Selected examples from scientific articles are discussed below to illustrate the feasibility of protein production under such conditions.

*Chlorella vulgaris* was cultivated in the 1-L glass photobioreactor having wastewater and incubation temperatures were modulated at the following three temperature regimes: low temperature (4 °C for 24 h) for 15 days, high temperature (35 °C for 24 h) for 15 days and high-low temperature (35 °C for 12 h: 4 °C for 12 h) for 15 days. It was found that switching high-low temperature (35 °C during at day and 4 °C at night) during cultivation process resulted in the highest biomass production (1.62 g·L^−1^). The protein contents were 24.2% and 18.4% at high temperature and low temperature conditions, respectively (Xu et al. 2019). A Danish strain of *Scenedesmus sp.* cultivated under ambient temperature and sunlight in tubular photobioreactors provided an average biomass productivity of 0.083 g DM·L⁻¹. The biomass contained 52.4% protein with EAAs accounting for 42% of total protein (Olsen et al. 2021).

*Concentrations of organic and inorganic carbons*

**Fig. 3:**
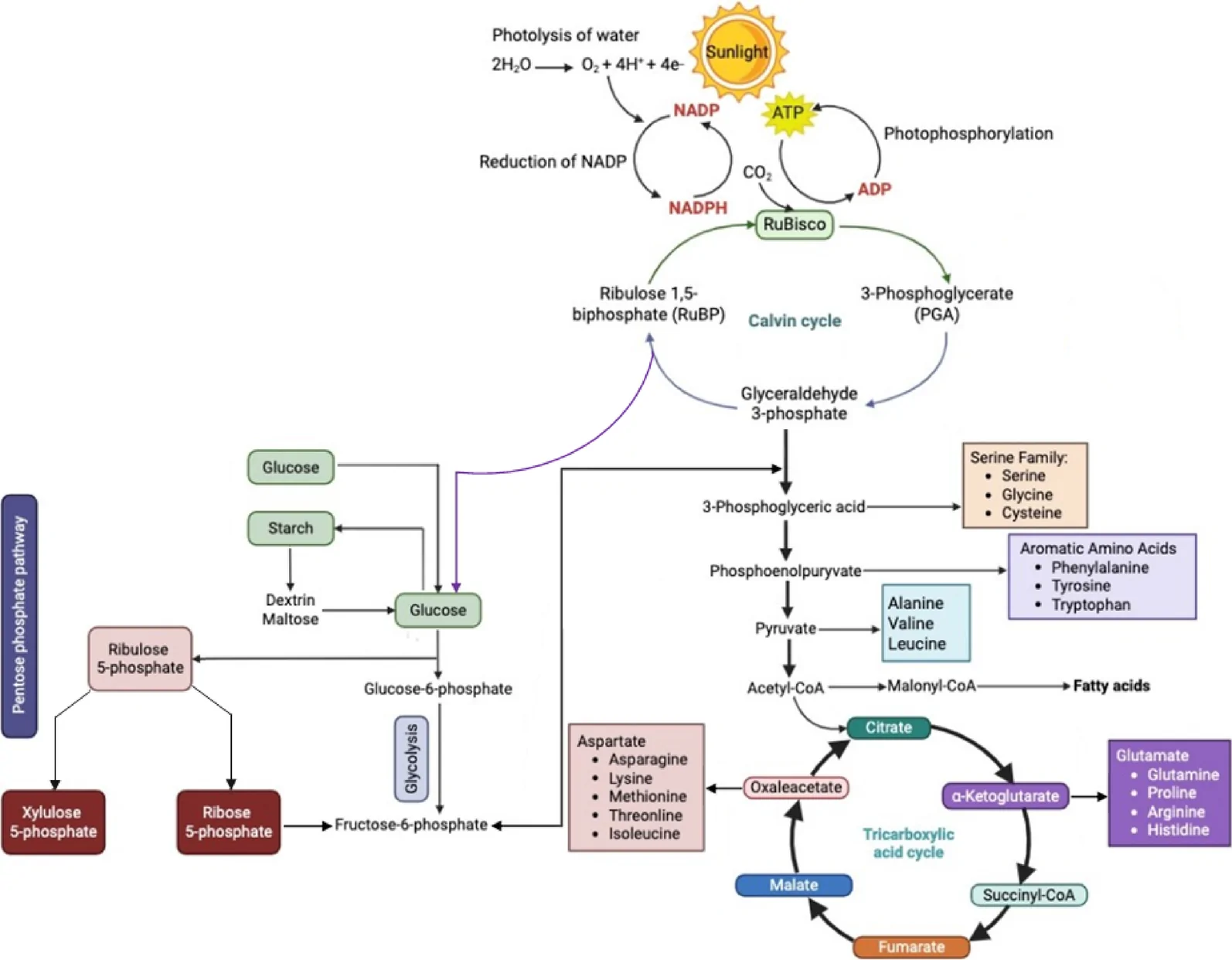
Metabolic pathways involved in protein biosynthesis in microalgae utilizing inorganic and organic carbon sources under photoautotrophic and heterotrophic growth conditions, respectively. In photoautotrophic growth, inorganic carbon (CO₂) is fixed via the Calvin-Benson-Bassham cycle into glyceraldehyde-3-phosphate, which acts as a central metabolic intermediate. Glyceraldehyde-3-phosphate is subsequently converted into glucose-6-phosphate and glucose-1-phosphate, supporting the biosynthesis of storage carbohydrate starch. In parallel, these intermediates enter glycolysis, generating pyruvate along with key metabolic precursors including 3-phosphoglycerate and phosphoenolpyruvate. Pyruvate is further converted into acetyl-CoA, which enters the tricarboxylic acid cycle, producing intermediates, such as citrate, α-ketoglutarate, succinate, fumarate and oxaloacetate. Collectively, intermediates from glycolysis and the tricarboxylic acid cycle, including α-ketoglutarate and oxaloacetate, function as essential carbon skeletons for amino acid biosynthesis. Under heterotrophic conditions, microalgae utilize organic carbon sources, such as glucose, which are metabolized through glycolysis and the pentose phosphate pathway. Glucose is first phosphorylated to glucose-6-phosphate and further converted into intermediates such as ribulose-5-phosphate. Ribulose-5-phosphate is subsequently transformed into ribose-5-phosphate and xylulose-5-phosphate, which are rearranged to form glycolytic intermediates, including fructose-6-phosphate and glyceraldehyde-3-phosphate, thereby linking the pentose phosphate pathway to glycolysis. These intermediates are further metabolized to generate energy and carbon skeletons required for amino acid and protein biosynthesis. (self-developed, concept was adopted from Xiao et al. (2022) and Ma et al. (2020)

Microalgae usually consume CO₂ from the environment for their photoautotrophic growth, whereas they consume organic carbon for their heterotrophic and photoheterotrophic growth. Under mixotrophic growth conditions, both inorganic and organic carbons are utilized by microalgae. CO₂ is reduced to glyceraldehyde 3-phosphate (G3P) in stroma of chloroplasts by ATP and NADPH produced in the thylakoid membranes of chloroplasts. G3P can be converted to glucose, the building block of starch through photosynthesis in chloroplast. Under stress conditions, starch could be converted to glucose and subsequently transformed to G3P in cytosol of microalgae. Amino acids, the building block of proteins, are produced when G3P could be converted to pyruvate (Pyr) and in the TCA cycle. For example, serine, glycine and cysteine are produced from 3 phosphoglyceric acid; tryptophan, tyrosine and phenylalanine are produced from phosphoenolpyruvate; alanine, valine and leucine are produced from Pyr. α ketoglutarate (αkg) and oxaloacetate (Oxa) in TCA cycle play a pivotal role for amino acid biosynthesis through the formation of glutamate and aspartate. Glutamine, proline, arginine and histidine are produced from glutamate. Aspartate could be converted to asparagine, lysine, methionine, threonine and isoleucine (Tibocha-Bonilla et al. 2018). Similarly, when organic carbon (glucose) enters the cytosol of microalgae, it could be oxidized via glycolysis or pentose phosphate pathway (PPP). In the glycolysis pathway, organic carbon could be converted to G3P through the formation of glucose 6-phosphate (G6P) and fructose-6P, and subsequently it is converted to Pyr, αkg and Oxa. On the other hand, glucose could be converted to xylulose 5-phosphate and ribose-5 phosphate via ribulose 5-phosphate formation, and subsequently converted to G3P (Ma et al. 2020; Xiao et al. 2022). Proteins and protein-complexes in microalgal cells are located in different parts, such as the cell wall, cytoplasm and cell organelles (Safi et al. 2015). Metabolic pathways for the biosynthesis of proteins from inorganic and organic carbons in microalgae are presented in Fig. 3.

Microalgae are able to grow within a wide range of CO_2_ concentrations in the environment and their CO_2_ fixation rates are 5–8 times higher than terrestrial plants. Pure CO_2_ is less water soluble, which limits carbon capture and fixation rate by microalgae (Ibrahim Mze et al. 2023). The concentration of environmental CO_2_ and the pH of the cultivation medium have important aspects on CO₂ assimilation by microalgae, thereby protein accumulation because the increase of environmental CO_2_ leads to a decrease in the pH of the cultivation medium (Singh and Singh 2014).

The different growth characteristics of microalgae based on energy and carbon source, such as photoautotrophy, heterotrophy, photoheterotrophy and mixotrophy are associated with microalgal protein and biomass yield (Young et al. 2022). Carbon flux balance analysis has been widely used to understand the genome-wide metabolic flux distributions under photoautotrophy, heterotrophy, photoheterotrophy and mixotrophy microalgal growth conditions. Studies at both transcriptomic and proteomic levels have demonstrated that central metabolic gene expression patterns including Calvin cycle, glycolysis, PPP and TCA cycle are differently modulated depending on the predominant growth characteristics of microalgae (Stavridou et al. 2024). Controlled stress conditions, such as nutrient availability and the modulation of light intensity have also been shown to play a decisive role in redirecting metabolic fluxes toward protein biosynthesis, accumulation of specific amino acids and overall biomass growth (Kaur et al. 2025). Summarized information about different types of algal growth and their characteristics is mentioned in Table 2.

**Table 2:** Characteristics of algal growth depending on culture conditions (self-developed, concept was adopted from Kamalanathan et al. (2018)

Culture type	Energy source	Carbon source
Photoautotrophy	Light (Obligatory)	Inorganic carbon (CO_2_)
Heterotrophy	Organic carbon. No requirement of light	Organic carbon glucose
Photoheterotrophy	Light (Obligatory)	Organic carbon
Mixotrophy	Light and organic carbon, but light not obligatory	Inorganic and organic carbon

The photoautotroph cultivation strategy is the most economical and comparatively easy for the production of microalgae; however, the growth of microalgae and quantity of protein obtained through this process is not often appreciable because the autotrophy metabolism is dependent on CO_2_ supply and light source. In autotrophic cultivation strategy, light intensity and photoperiod critically modulate carbon fixation rate and photosynthetic efficiency, nitrogen assimilation and biomass productivity, thereby directly influencing protein biosynthesis in microalgae. In photosynthesis, suboptimal light wavelength restricts the generation of ATP and NADPH; whereas, excessive light wavelength may cause oxidative stress and photoinhibition, resulting in reduced protein biosynthesis. Furthermore, photoperiod modulates circadian-mediated metabolic processes with controlled light-dark cycles enhancing protein content and biomass productivity (Rivera-Sánchez et al. 2025). Unlike the utilization of CO_2_ in autotrophy metabolism, the heterotrophic metabolism, an aerobic process, is dependent on organic carbon sources, such as glucose, glycerol and organic acids which satisfy the substrate and energy needs of microalgae. The organic carbon in the heterotrophic metabolism can be supplied in the form of carbohydrate (glucose and saccharified lignocellulosic sugars), glycerol, wastewater having organic load, and so on. Organic carbons produce energy through the oxidative phosphorylation accompanied by O_2_ consumption as the final electron acceptor in the heterotrophic metabolism of microalgae. The development of a heterotrophic technology is expected to be the most suitable option, because high concentration of algal biomass could be produced within a photobioreactor, which is independent of the light source and its environmental impacts are usually lower (Xu et al. 2021; Jin et al. 2021; Xiao et al. 2022); however, the cultivation of a heterotroph process may have significant expenses due to organic carbon source in the cultivation medium, and accessories of bioreactor. Furthermore, there is a high chance for contamination of cultures by heterotrophic fungi and bacteria (Scott et al. 2010). The photo-heterotrophic cultivation strategy is a rarely used approach where organic carbon and light are prerequisite for the growth of microalgae as it integrates both phototrophic and heterotrophic metabolisms. At the genomic level, photo-heterotrophic cultivation strategy coordinates the expression of genes involved in photosystem function, light-harvesting complexes, glycolysis, the TCA cycle and nitrogen assimilation. Moreover, stress-response and antioxidant genes are activated to mitigate ROSs produced from simultaneous photosynthetic and respiratory activity in photo-heterotrophic cultivation strategy. The levels of ROSs are influenced by inorganic carbon-driven photosynthesis, organic carbon-supported respiration, light intensity-driven electron transportation and photoperiod-determined photosynthetic activity duration. Therefore, the dual uses of light and organic carbon source allow cells to optimize metabolic fluxes towards protein biosynthesis (Wang et al. 2014a). In order to reduce the mentioned limitations of single cultivation strategy, sequential heterotrophic-autotrophic (Ogbonna et al. 1997; Fan et al. 2012; Barros et al. 2019) and mixotrophic (Matos et al. 2017; Abiusi et al. 2022; de Mendonça et al. 2022) cultivation systems were employed in some cases. Inorganic and organic carbons are simultaneously utilized in the presence of light in the mixotrophic metabolism, where CO_2_ is fixed through photosynthesis in the presence of light (Photoautotrophic metabolism), while organic carbon is assimilated through the aerobic respiration (Heterotrophic metabolism) (Wang et al. 2014a). In mixotrophic cultivation strategy, light intensity and photoperiod play a significant role by supporting photosynthetic activity and indirectly sustaining respiratory metabolism through improved cellular energy generation and redox balance. Moderate light intensities in mixotrophic culture condition often enhance the biosynthesis of protein by improving redox-balance and energy availability compared to stringently autotrophic culture condition. Extended photoperiods in mixotrophic cultivation system can promote the biosynthesis of protein by sustaining photosynthetic ATP and NADPH generation which are critical for anabolic processes, maintaining redox balance and regulating the expressions of photosynthesis-associated genes. Moreover, extended photoperiods in mixotrophic cultivation system simultaneously enhances respiratory metabolism or the efficiency of organic carbon assimilation by supporting the transcription of genes involved in carbon transportation, nitrogen assimilation and central metabolic pathways (glycolysis and the TCA cycle), thereby improving protein biosynthesis and biomass productivity (Matos et al. 2017; Abiusi et al. 2022; de Mendonça et al. 2022). Several microalgae species are able to switch between photo-autotrophic and heterotrophic metabolisms, which is not similar to mixotrophic metabolism, where both organic and inorganic carbons utilization occur simultaneously (Ogbonna et al. 1997; Fan et al. 2012; Barros et al. 2019). It may be realized that the combined cultivation process could be quite attractive because the disadvantages of each cultivation system may be covered up and a host of advantageous opportunities may be explored.

*Concentration of nitrogen*

Nitrogen is the second most important substrate after carbon source for the growth of microalgae and it is the precursor of wide ranges of metabolites including protein. Microalgae have evolved a variety of biochemical mechanisms and regulatory systems for assimilating inorganic and organic nitrogenous compounds from the culture medium. The correlation between nitrogen concentrations and protein productivity in microalgae is essential to comprehend because nitrogenous compounds are strongly related with cellular physiology. In most cases, it was observed that the protein content in microalgae could significantly increase with increasing initial nitrogen concentration in the cultivation medium; however, protein content in microalgae could significantly reduce with more nitrogenous items in the cultivation medium (Suttle et al. 1990; Kudelala and Cochlan 2000). Microalgae are able to utilize various forms of nitrogen sources, including inorganic nitrogen sources, such as nitrite, nitrate and ammonium, and organic nitrogen source, such as urea (Kumar and Singh 2019). In microalgae, both nitrate and nitrite enzymes act as repressible enzymes where nitrate enhances the physiological activity of cells. On the other hand, the function of ammonium in microalgae is versatile (Terrado et al. 2015). Various transporter proteins and permease enzymes are involved in the uptake of miscellaneous nitrogen sources to microalgal cells. The differential capacities of green microalgae to utilize nitrogenous compounds arise from variations in the availability of transport systems and catabolic enzyme machinery (Flores and Herrero 2005; Hellebust and Ahmad 2013).

*Ammonium and ammonia*

**Fig. 4:**
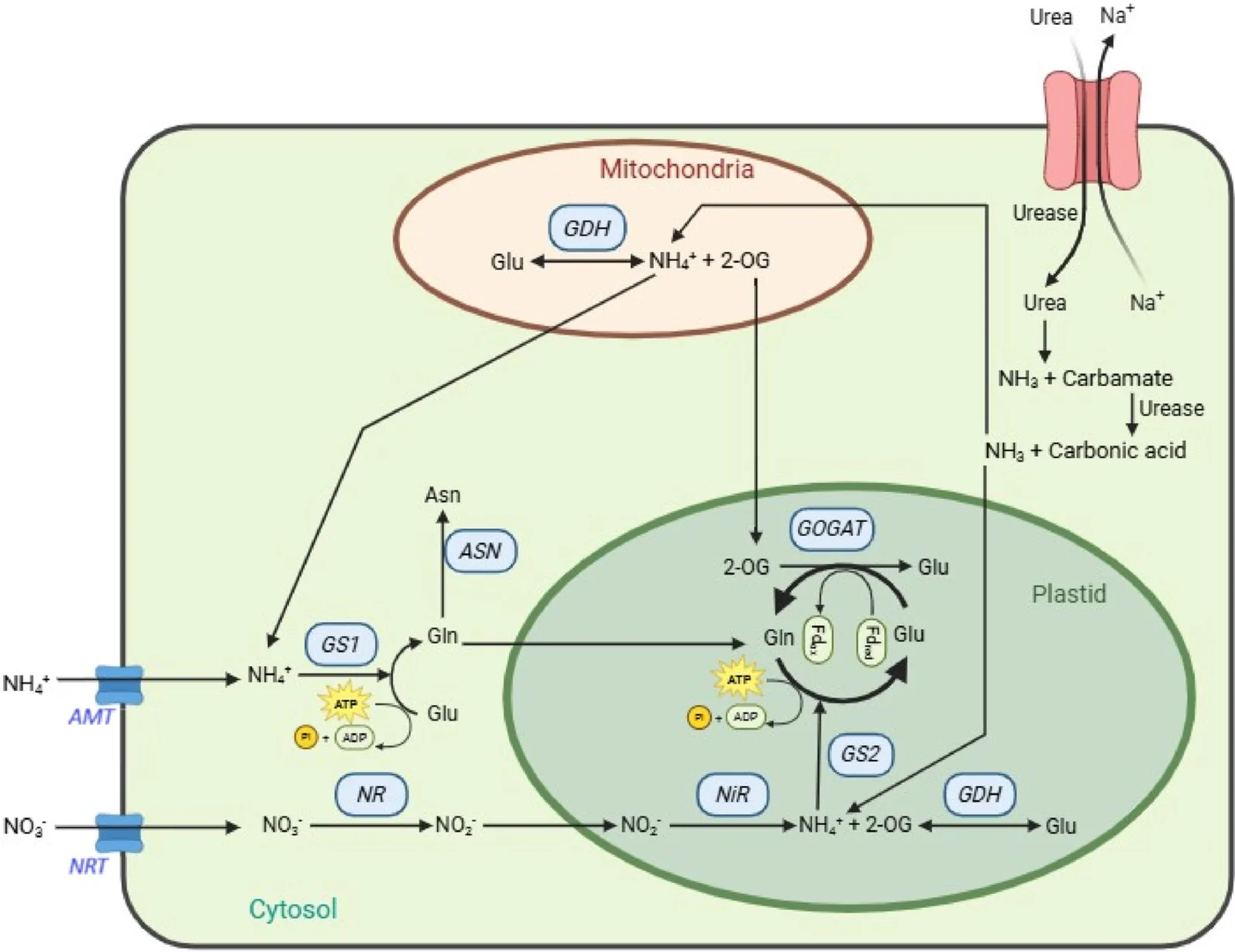
Metabolic pathway for the assimilation of nitrogen sources in microalgae. The uptake of ammonium (NH_4_^+^) and nitrate (NO_3_^–^) ions is mediated by ammonium transporter (AMT) and nitrate reductase transporter (NRT), respectively. Ammonium ion is transported by ammonium transporters and assimilated into organic compounds via the glutamine synthetase-glutamate synthase (GS-GOGAT) cycle by the action of glutamine synthetase (GS) and glutamate synthase (GOGAT). Enzymes glutamate dehydrogenase (GDH) and asparagine synthetase (ASN) also participate in ammonium assimilation. The NO_3_^–^ is entered into the cell and is reduced to nitrite ions (NO_2_^–^) by nitrate reductase (NR). Subsequently, NO_2_^–^ is transported to plastid and reduced to ammonium ion by nitrite reductase (NiR). These biochemical pathways play key roles in the synthesis of glutamine (Gln), glutamate (Glu) and asparagine (Asn) (self-developed, concept was adopted from Kumar and Bera (2020) and Goel and Singh (2015)

Ammonium and ammonia are two of the most preferential among inorganic nitrogen sources for microalgae and their interchange depends on the pH and the CO_2_ concentration in the cultivation medium. Higher aeration rate could assist air stripping of ammonia from non-volatile ammonium ions (NH_4_^+^) dissolved in the cultivation medium. Ammonium has no effect on nitrate reduction in carbon deprived algal cells (Cao et al. 2019). Ammonium, which is a preferred nitrogen source under most conditions, is usually assimilated via the GS-GOGAT-mediated pathways where the α-ketoglutaric acid from the mitochondrion is transformed to glutamine by glutamine synthetase and GOGAT isoenzymes catalyze the biotransformation of the amido nitrogen of glutamine to 2-oxoglutarate using NADPH or ferredoxin as reductants (Fig. 4). Therefore, irrespective of phototroph or heterotroph metabolism, a part of the Krebs cycle maintains the carbon flow within the cell (Weger et al. 1988). It is important to mention that the expression of GS in *Chlorella vulgaris* was up-regulated 6.4-fold when the ammonium concentration was under 10 mg·L^−1^ compared to 4 mg·L^−1^ which implies the key role of GS for the assimilation of ammonium in the mentioned microalgae (Liu et al. 2015). Furthermore, ammonium is assimilated via NADP-dependent glutamate dehydrogenase (GDH) pathway in some microalgae (Fig. 4). *Stichococcus bacillaris* possesses a high-affinity NADP-GDH under restricted ammonium supply (Ahmad et al. 1986); whereas, *Chlorella* sp. possesses a high-affinity NADP-GDH under higher ammonium conditions (Tischner 1984). *Stichococcus bacillaris* (Ahmad et al. 1986) and *Chlamydomonas reinhardii* (Cullimore and Sims 1981) retain both a ferredoxin (Fd)- and NAD-dependent GOGAT pathways, which are linked to the assimilation of ammonium in the presence and absence of light, respectively. The activity of the GS-GOGAT pathway is superior in cells during the initial part of the light phase, and eventually NADP-GDH becomes preferential for the assimilation of ammonium (Tischner and Hüttermann 1980). The NADP-dependent glutamate dehydrogenase activity primarily appeared under heterotrophic condition (Prunkard et al. 1986) and when amino acids were the sources of nitrogen in cultivation medium (García-Domínguez et al. 1997). Microalgae do not consume other nitrogen sources until all ammonium is utilized (Florencio and Vega 1983; Fernandez and Galvan 2007); however, some scientists do not agree with such a universal statement for the complexity of nitrogen assimilation by strains (Gutierrez et al. 2016). Uptake of ammonium takes place much more rapidly in the presence of light than in the absence of light when cells are grown under nitrogen-sufficient conditions; however, uptake of ammonium could be rapid in the absence of light when cells have low nitrogen content but high carbon reserves (Grant and Turner 1969; Thacker and Syrett 1972). In microalgae, ammonium transporters located in the plasma and chloroplast membranes facilitate effective ammonium uptake and assimilation, thereby preventing the utilization of alternative nitrogen sources until all available ammonium is depleted (Fernandez and Galvan 2007; Hellebust and Ahmad 2013). Methylammonium is a useful analogue for the transportation of ammonia in the biotic phase of microalgae. The activities of methylammonium/ammonium transport systems were reported for *Stichococcus bacillaris* (Ahmad and Hellebust 1985), *Chlamydomonas angulosa* (Hellebust et al. 1985), *Chlamydomonas reinhardii* (Xu and Pan 2020) and *Chiarella pyrenoidosa* (Wheeler 1980), unlike cyanobacteria (Kerby et al. 1987). Although ammonium is the preferred nitrogen source for microalgae, it can be toxic at high concentrations, resulting in growth inhibition and cell mortality (Li et al. 2019). Free unionized ammonia could interact with PS II proteins in microalgal cells and increase photosensitivity which leads to the damage of the photosystem (Markou et al. 2014). Furthermore, imbalanced concentration of ammonia could hinder the photophosphorylation process by reducing the pH gradient (Zhao et al. 2019). Ammonium assimilation may inhibit the transcription of mRNA for nitrate reductase synthesis, rather than ammonium alone being responsible for this effect in microalgae (Syrett and Leftley 2016). According to many investigators, this might be the outcomes of shared inhibitory influence of amino acids and the activity of nitrate reductase in nucleotides. Contradictorily, it could be merely the blockage of nitrate uptake by microalgal cells (Barros et al. 2017).

*Nitrate*

Assimilation of nitrate occurs when nitrate is reduced to nitrite followed by biosynthesis of ammonium/ammonia through sequential enzymatic (nitrate reductase and nitrite reductase) bioconversions (Fig. 4) (Bekheet and Syrett 1977). The basic processes of nitrification and denitrification in algae influence the assimilation of nitrogen oxides (nitrite or nitrate). The assimilation of nitrate and nitrite by microalgae is regulated at three levels: (a) the activity and capacity of nitrate and nitrite transport systems, (b) the activity of nitrate reductase and nitrite reductase, and (c) the amount of nitrate and nitrite reductases in the cells (Mandal et al. 2018). Nitrate and nitrite reductases are strongly regulated by light conditions and the nitrogen status of the cells. *Botryococcus braunii* and *Dunaliella tertiolecta* prefer nitrate as an inorganic N source; however, their growths are reduced in the presence of ammonium (Syrett and Leftley 2016). Interestingly, it was reported that the assimilation of ammonium by *Chlorella sorokiniana* is associated with the presence of light (Molin et al. 1981). In the presence of light, *Chlamydomonas* could grow with ammonium; however, the assimilation of nitrate or nitrite was strongly inhibited by ammonium (Florencio and Vega 1983). In another investigation, it was found that the regulation of oxidized nitrogen source utilization occurred only in blue light in *Monoraphidium braunii*, grown at pH 8 with blue and red light intermittently (Aparicio and Quiñones 1991).

*Urea*

**Fig. 5:**
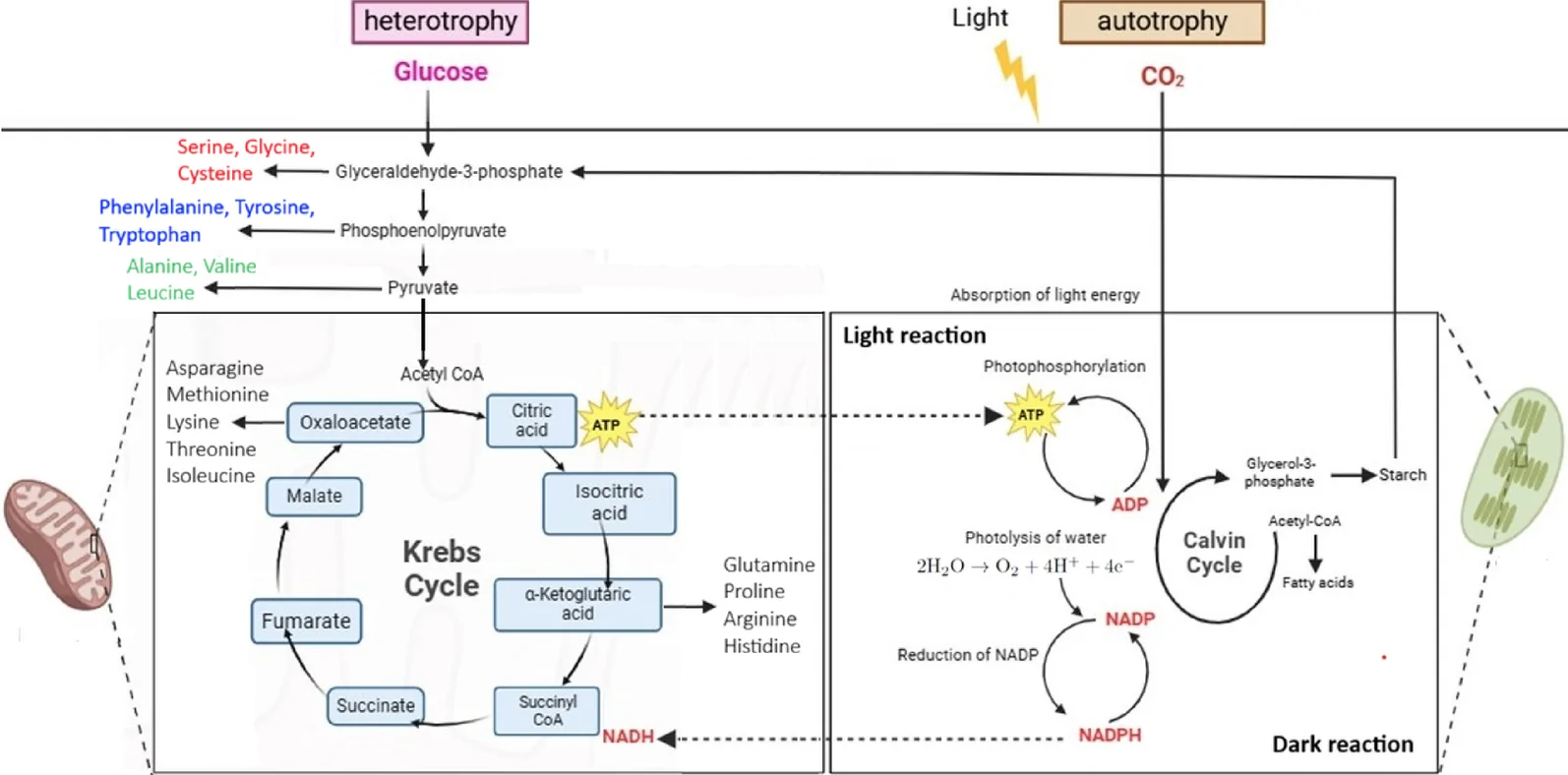
Metabolic pathways for sequential heterotrophic-autotrophic and mixotrophic growth of microalgae. In the autotrophic mode, sunlight acts as the sole energy source driving photosynthesis, where inorganic carbon (CO₂) is assimilated through the Calvin-Benson-Bassham (C₃) cycle. Ribulose-1,5-bisphosphate carboxylase/oxygenase (RuBisCO) catalyzes the carboxylation of ribulose-1,5-bisphosphate to form 3-phosphoglycerate, which is subsequently reduced to glucose using ATP and NADPH generated from the light-dependent reactions. The produced starch enters into glycolysis and the TCA cycle, supplying precursors amino acids of protein biosynthesis. Under heterotrophic growth, in the absence of light, microalgae rely entirely on external organic carbon (glucose) as both carbon and energy source. Glucose is metabolized via glycolysis to pyruvate, which enters the TCA cycle, providing ATP and reducing power for biosynthetic reactions. Mixotrophic growth integrates both pathways (autotrophic and heterotrophic), enabling simultaneous CO₂ fixation via the Calvin cycle and glucose catabolism through the glycolysis and the TCA cycle (self-developed, concept was adopted from Ogbonna et al. (1997), Fan et al. (2012), Barros et al. (2019), Matos et al. (2017), de Mendonça et al. (2022) and Abiusi et al. (2022)

The organic nitrogen compound urea is an essential nutrient for microalgal growth and is commonly found in various redox states in nature. Its uptake by algal cells typically occurs via ABC-type (ATP-binding cassette) transporters that use energy from ATP to transport urea across the cell membrane (energy-driven process). In addition to uptake of external urea into the cell, urea could be produced in the biotic phase by the urea cycle and/or by purine catabolism (BIRDSEY and LYNCH 1962; Borowitzka 2013). Once inside the cell, urea is deaminated to generate ammonium, a process catalyzed by urease or urea amidolyase. Urease catalyzes the hydrolysis of urea in two stages: in the first stage, urea is converted into ammonia and carbamate, and in the second stage, carbamate undergoes spontaneous hydrolysis to produce an additional ammonia molecule and carbonic acid (Fig. 5). Under physiological conditions, the dissociation of carbonic acid releases protons, which react with ammonia to form ammonium ions. Subsequently, ammonium ions can be assimilated by the microalgae to support their growth (NEILSON and LARSSON 1980; Veaudor et al. 2019). The presence of urease has been reported in several families of microalgae, including *Chlorellaceae* and *Pelagomonadaceae* (NEILSON and LARSSON 1980; Solomon et al. 2010). In microalgae and cyanobacteria, the urease activity and the size of the intracellular urea pool may be strongly influenced by several factors, such as the rate of urea uptake, the production of urea from the urea cycle and amino acid and purine catabolism (Leftley and Syrett 1973; Veaudor et al. 2019). In some microalgae, lower urease activity is found in NH_4_^+^-grown cultures than in NO_3_^–^- and urea-grown cultures, reflecting the preferential assimilation of ammonium as a readily available nitrogen source (Peers et al. 2000; Solomon et al. 2010). On the other hand, urease activity could be upregulated in urea-grown microalgae and can be further increased under nitrogen-limited conditions to enhance the utilization of available organic nitrogen sources (Solomon et al. 2010). The presence of NH_4_^+^ may inhibit the use of urea in many microalgae (NH_4_^+^ repression) (Solomon et al. 2010). The second enzyme, urea amidolyase, consists of two functional domains: urea carboxylase and allophanate hydrolase. The urea carboxylase is a biotin-dependent enzyme that catalyzes the carboxylation of urea through the sequential action of biotin carboxylase and carboxyltransferase domains, utilizing ATP to produce allophanate. Subsequently, the nitrogen and carbon domains of allophanate hydrolase act sequentially on allophanate to generate ammonia and carbonic acid (Solomon et al. 2010).

#### Process operational strategy

Process operational strategy plays a critical role in determining the performance of microalgal growth, metabolic activity and protein yield. In microalgal bioprocesses, operational strategy refers to the selection of cultivation modes which is associated with available carbon (inorganic or organic) and other nutrient sources, and light conditions. From the process intensification perspective, the design of an appropriate operational strategy is essential to improve the growth of microalgae and the protein yield along with the maximized resource utilization, enhanced metabolic efficiency and by-product utilization within a lowered cultivation time and reactor footprint. Microalgae possess notable metabolic flexibility and can grow under different trophic cultivation systems. Each cultivation system influences carbon assimilation metabolic pathways, cellular energy generation and the regulation of metabolic fluxes for protein and other metabolites biosynthesis. Operational parameters, such as light intensity and photoperiod, temperature and bioavailable carbon and nitrogen sources strongly affect photosynthetic performance, respiratory metabolism, nitrogen assimilation and redox balance in microalgal cells. Advanced cultivation approaches, including sequential heterotrophic-autotrophic cultivation system (Ogbonna et al. 1997; Fan et al. 2012; Barros et al. 2019), mixotrophic cultivation system (Matos et al. 2017; Abiusi et al. 2022; de Mendonça et al. 2022) and switching of nitrogen-rich cultivation medium from nitrogen-deficient medium (Xie et al. 2017; Dasan et al. 2021; Xiao et al. 2022; Liang et al. 2023) have been explored to overcome the inherent limitations of single cultivation strategy by integrating the different metabolic pathways towards protein biosynthesis. Such integrated operational strategies can improve carbon utilization efficiency, support higher cell densities and promote the protein yield. From the bioprocess engineering perspective, the integrated strategy provides operational flexibility and fine-tuning of operational parameters by permitting independent optimization of cultivation conditions in each stage, resulting in improvement of protein productivity from microalgae. Therefore, the selection of an appropriate cultivation strategy along with suitable operational parameters is a fundamental aspect of process intensification for the efficient production of protein from microalgae.

*Sequential heterotrophic and autotrophic cultivation*

Two-stage of cultivation strategy was considered to obtain higher protein yields from microalgae, involving sequential heterotrophic and autotrophic cultivation strategies. In heterotrophic cultivation system (independent of light availability), microalgae proficiently assimilate organic carbon through respiratory metabolic pathways, which promotes the productivity of biomass. Due to light independent strategy, light-induced stress followed by oxidative damage of photosynthetic machinery in the growth phase of microalgae is absent in heterotrophic cultivation system. Following the formation of biomass, a shift to autotrophic condition activates light-driven photosynthetic metabolism, allowing electron transport to generate ATP and NADPH that support glycolysis, TCA cycle and nitrogen assimilation pathways including GS-GOGAT cycle, while central metabolic pathways supply precursor molecules for protein biosynthesis. By separating growth and protein biosynthesis phases, the two-stage cultivation strategy promotes overall carbon and nitrogen assimilation by microalgae and subsequently improves protein productivity (Ogbonna et al. 1997; Fan et al. 2012; Matos et al. 2017; Barros et al. 2019; Abiusi et al. 2022; de Mendonça et al. 2022). Metabolic pathways for sequential heterotrophic-autotrophic and mixotrophic growth of microalgae are presented in Fig. 5.

Investigations have been performed with both laboratory-scale and industrial-scale setups to understand and optimize the cultivation parameters of the sequential heterotrophic-autotrophic metabolisms of microalgae for maximizing protein yield. The following section provides information about recent studies on sequential heterotrophic and autotrophic cultivation strategies for the production of protein from microalgae. Under the two-stage cultivation strategy, biomass productivity and biosynthesis of protein are influenced by several operational parameters, such as light wavelength, light intensity, photoperiod, and the type and concentration of carbon and nitrogen sources. Since the optimum cultivation parameters vary between the heterotrophic and autotrophic growth stages of microalgae, independent optimization of process parameters of each cultivation stage is prerequisite for maximizing biomass productivity and protein yield from microalgae. The protein productivity of microalgae for these cultivation conditions is highly strain-specific because each strain possesses unique metabolic characteristics and regulatory mechanisms. Therefore, making comparisons among studies is considerably challenging.

Batch-mode heterotrophic cultivation of *Chlorella pyrenoidosa* C-212 was performed in a basal medium having 28 g·L^−1^ of glucose. Subsequently, the culture broth was transferred into a Roux-flask for the autotrophic growth when the glucose concentration was reduced to zero in heterotrophic cultivation system. In photoautotrophic cultivation system, the light intensity at the surface of the flask was maintained 310 µmol photon·m^−2^·s^−1^. Aeration and mixing were maintained by sparging sterile air having 5% CO_2_ through a glass-ball filter with a constant flow rate of 0.3 vvm. The cellular protein content of microalgae was increased gradually from 30 to 60% (dry basis) over a 180-day cultivation period and the rise continued even after 96 h of autotrophic cultivation. In the later exercise, continuous mode heterotrophic cultivation was performed in a stirred fermenter having a working volume of 2.0 L. The cultivation temperature and aeration rate were maintained similar to batch-mode cultivation process; however, agitation speed was maintained 300 rpm. The continuous feeding of 5 times concentrated basal medium containing glucose (35 g·L^−1^) was started with a flow rate of 15 mL·h^−1^ when the glucose concentration was reduced to zero. The effluent from the heterotrophic culture medium was transferred to the autotrophic cultivation system, taking place in a tubular photobioreactor having working volume 450 mL. The average residence time of the culture broth in the tubular photobioreactor was maintained at 30 h. The incubation temperature and light illumination of the autotrophic phase were maintained similar to the batch-mode cultivation process. The exhaust gas from the heterotrophic cultivation system was used for aeration in the tubular photobioreactor through a glass-ball filter with a constant flow rate of 0.5 vvm. The protein content of the cells in the effluent of the autotrophic phase was increased to very high values of 60.1 at the steady state of the photobioreactor (Ogbonna et al. 1997). In another investigation, a two-stage scalable cultivation process was developed with the aid of higher protein yield and biomass concentration. *Chlorella vulgaris* was cultivated in heterotrophic condition in a 200-L and 5000-L fermenters sequentially (1st stage) and culture from 5000-L fermenter was used directly as inoculum to outdoor eight industrial-scale tubular photobioreactor having a working volume of 100 m^3^ each (2nd stage) where autotrophic culture condition was maintained. Heterotrophic culture medium was composed with glucose as the organic carbon source and nitrate as a N source maintaining a C/N ratio 6.7:1, pH 6.5. The bioreactor was operated in fed-batch mode as glucose was fed upon its depletion and the incubation temperature was maintained at 28 °C. The air inlet flow rate was maintained ~ 1 vvm and the agitation rate ranged from 100 to 1200 rpm throughout the heterotrophic cultivation period. The autotrophic culture condition was maintained to a natural circadian light : dark cycle (16 h of light/ day and peak radiation 687 W·m^−2^). The pH of culture medium in photobioreactor was maintained within 5.5 and 6.5 by injecting pure CO_2_ and continuous aeration. The maximum biomass concentration after 4 days of heterotrophic cultivation was 120 g·L^−1^ in both 200-L and 5000-L fermenters. The biomass concentration was increased from 0.75 g·L^−1^ (initial biomass concentration of inoculum in photobioreactor) to 1.09 g·L^−1^ in photobioreactor after 14 days of cultivation. The concentration of protein in the photobioreactor after seeding from the heterotrophic cultivation system was increased from 32 to 52.18% of dry weight after 14 days of cultivation (Barros et al. 2019).

*Sequential nitrogen starvation followed by exposure to nitrogen-rich condition*

A two-stages of cultivation strategy, such as sequential nitrogen starvation followed by exposure to nitrogen-rich conditions, was adopted to achieve a higher protein yield from the biomass of microalgae. During the first nitrogen-starvation stage, microalgae experience a strong metabolic shift that suppresses nitrogen assimilation and protein biosynthesis while carbon storage pathways, such as carbohydrate and lipid accumulations are upregulated. This phase enables higher biomass formation and cellular restructuring, as the limitation of nitrogen reduces energy allocation toward ribosomal activity and the biosynthesis of amino acids. Furthermore, nitrogen starvation induces global transcriptional readjustment, resulting in the downregulation of nitrogen-assimilation involving genes and the upregulation of stress-responsive genes. Therefore, this strategy modulates overall carbon-partitioning metabolic pathways. During the nitrogen starvation period, these metabolic changes prepare cells for rapid nitrogen assimilation by increasing the responsiveness of nitrogen transporter and assimilation pathways. In the second stage, nitrogen supply rapidly activates transporters of nitrate and ammonium, upregulates nitrate reductase and influences the GS-GOGAT pathway, resulting in accelerated assimilation of nitrogenous compounds from the culture medium. A sudden shift of the bioavailability of nitrogenous compounds stimulates biogenesis of ribosomal proteins, translational activity and the biosynthesis of amino acids, resulting in a sharp increase of protein formation. In photoheterotrophic, heterotrophic and mixotrophic cultivation systems, the exposure in nitrogen-enriched medium after a nitrogen starvation phase offers advantages, such as availability of nitrogen and energy from carbon metabolism to microalgae that upregulates protein biosynthetic fluxes. Furthermore, nitrogen resupply stimulates the mobilization of carbon reserves taken during the nitrogen starvation period towards the biosynthesis of amino acids via the upregulation of central carbon metabolic pathways (Xie et al. 2017; Dasan et al. 2021; Xiao et al. 2022; Liang et al. 2023).

Nitrogen starvation (first stage) followed by exposure of microalgae in a nitrogen-rich source (second stage) in the continuous mode operation has been employed to obtain a higher yield of protein from *Graesiella emersonii* WBG-1, isolated from Chenghai Lake in southwest China. A constant feeding rate of medium (nitrate-free) 0.52 mL·min^−1^ was maintained which yielded the growth rate of microalgae 0.96 d^−1^. When the first stage of the cultivation system with the initial nitrogen concentration 5.88 mM was reached to steady-state, the output biomass was cultivated in another cultivation medium (cultivation medium enriched with nitrate) operated with steady-state. It was found that nitrogen content in microalgal biomass was reduced by the stepwise reduction of nitrate concentration in the feeding medium (9.4–0.2 mM). Investigators concluded that when light illumination, temperature and the flow rate of CO_2_-enriched sterile air in both steps of cultivation systems were constant, *Graesiella emersonii* is able of growth for days independent of abiotic nitrogen, and only 30% of saturated nitrogen availability and 47% of maximum protein content are required to maintain a continuous operation of chemostat with growth rate of microalgae 0.96 d^−1^ (Liang et al. 2023). In another investigation, two stages of the batch-mode cultivation of *Chlorella sorokiniana* were performed with different concentrations of glucose and urea. In the first stage, the cultivation medium was composed with higher ratio of glucose and urea; whereas, in the second stage of the cultivation process, a nitrogen rich cultivation medium containing glucose (higher ratio of urea and glucose) was used. In a later experiment, the similar concept (higher ratio of glucose and urea in first stage of cultivation and higher ratio of urea and glucose in second stage of cultivation) was considered when the bioprocess was performed with fed-batch operating mode. Protein concentration was increased from 20 g·L^−1^ in the first-stage of cultivation process to 65 g·L^-1^ in the two-stage cultivation process. Investigators concluded that higher ratio of glucose to urea in the first stage of cultivation process could improve the biomass growth and intracellular starch concentration instead of the intracellular protein content; however, it could be switched in opposite way due to higher ratio of urea to glucose in the second-stage of cultivation process (Xiao et al. 2022). Similar outcomes were also published by Xie with co-authors for *Chlorella vulgaris* (Xie et al. 2017).

*Mixotrophic cultivation strategy*

The mixotrophic cultivation strategy represents an attractive approach for scalable microalgal growth and the biosynthesis of protein by integrating the simultaneous utilization of CO_2_ through light-driven photosynthesis and organic carbon through respiratory metabolic pathways. In this integrated metabolic system, light-driven photosynthesis assimilates CO_2_ and generates ATP and NADPH, which support key metabolic processes, including carbon fixation and nitrogen assimilation pathways such as the GS-GOGAT cycle. In parallel, organic carbon is metabolized through glycolysis and the TCA cycle, providing additional energy and carbon skeletons required for amino acid synthesis under mixotrophic conditions. Therefore, mixotrophic growth conditions induce the coordinated expression of genes involved in light-driven photosynthesis, carbon transport, nitrogen assimilation and central carbon metabolism (glycolysis and the TCA cycle). Therefore, it improves the overall cellular energy status, reduces stress-induced growth arrest and dependency on light as the sole energy source, and mitigates carbon limitation by maintaining continuous substrate bioavailability. Mixotrophic cultivation strategy could provide higher protein yield compared to strictly autotrophic or heterotrophic cultivation strategy (Matos et al. 2017; Abiusi et al. 2022; de Mendonça et al. 2022).

Mixotrophic metabolism is characterized by the interconnected metabolisms of both phototrophic and heterotrophic pathways, consenting microalgae to utilize light energy and organic carbon sources simultaneously. Therefore, biosynthesis of protein in microalgae under mixotrophic condition is influenced by several factors, including light wavelength, light intensity, photoperiod, and the type and concentration of carbon and nitrogen sources. The outcomes of recent studies about the effects of different mixotrophic cultivation parameters on microalgal growth and protein production are mentioned herein. Since these parameters regulate both metabolic pathways simultaneously, their individual effects on biomass productivity and protein biosynthesis are often challenging to differentiate. Furthermore, the physiological responses of microalgae to these cultivation parameters are varying strain to strain because each species has unique metabolic characteristics and regulatory mechanisms; thereby, the optimal process parameters of cultivation condition stated for a microalgal strain cannot be directly inferred to another, making comparisons among studies particularly challenging.

*Nannochloropsis gaditana* was cultivated in mixotrophic strategy in 75% desalination concentrate with 2.0 g·L^−1^ glucose under a light intensity 100 µmol photon·m^−2^·s^−1^ by four 20 W cool white fluorescent lamps. It was found that maximum cell growth and protein production were photoperiods 16 : 08 L : D and 12 : 12 L : D, respectively (Matos et al. 2017). *Scenedesmus obliquus* was cultivated in two identical vertical alveolar panel photobioreactors among them, one of the vertical alveolar panel photobioreactors was attached to CO_2_ pressurized cylinder. The untreated wastewater from a confined cattle stable facility in the region of Loures, Portugal was considered for the culture medium of *Scenedesmus obliquus* and the temperature of the cultivation process was maintained 18–21 °C. The continuous illumination at an average light intensity of 58 µmol·m^−2^·s^−1^ was supplied by four 58 W cool-white lamps in cultivation processes. However; air injections having 0.044% CO_2_ were considered with flow rate of 2.6 L·min^−1^ for both photobioreactors, CO_2_ flow with flow rate of 0.008 L·min^−1^ was used as a photobioreactor to maintain the pH of the culture medium 7. Protein productions from *Scenedesmus obliquus* by batch and continuous modes were 32% and 53%, respectively without additional CO_2_ supply; whereas, protein productions from *Scenedesmus obliquus* by batch and continuous modes were 42% and 55% with additional CO_2_ supply, respectively (de Mendonça et al. 2018). In another investigation, *Chlorella vulgaris* and *Scenedesmus obliquus* were cultivated separately in two identical vertical alveolar panel photobioreactors by batch-mode and continuous-mode cultivation strategies. Synthetic dairy wastewater was considered as a cultivation medium of microalgae and the temperature of the cultivation process was maintained 23 °C. The continuous illumination at an average light intensity of 60 µmol·m^−2^·s^−1^ was supplied by four 58 W cool-white lamps in cultivation processes. Air injections having 0.044% CO_2_ were considered with flow rate of 1.6 L·min^−1^ were used to maintain the pH of the culture medium 7 and 7.5. Protein productions from *Chlorella vulgaris* by batch and continuous modes were 40% and 53%, respectively; whereas, protein productions from *Chlorella vulgaris* by batch and continuous modes were 5% and 56% (average value for 7 days cultivation), respectively (de Mendonça et al. 2022). Mixotrophic cultivation of *Galdieria sulphuraria* in Zarrouk medium, pH 9.2 was performed in a stirred-tank bioreactor. The temperature and an average photon flux density were maintained 37 °C and 511 µmol·m^−2^·s^−1^, respectively. For autotrophic operation condition, the reactor was sparged with air enriched with 2% v/v CO_2_ at a flow rate of 1 L·min^−1^. The stirred-tank bioreactor was inoculated with an initial biomass concentration of 1.25 g·L^−1^ and the cultivation was performed with batch mode until a biomass concentration was reached to 4 g·L^−1^ after which the system was operated in autotrophic chemostat mode with a dilution rate 0.2 day^−1^. Subsequently, the bioreactor was operated with a batch mode for 1.5 days with constant glucose addition (10% w/w glucose solution was added at a rate of 3.7 g·h^−1^) and sparging 2% CO_2_ enriched air. After this period of adaptation to glucose, aeration was switched off and the oxygen-balanced mixotrophy culture condition was maintained by controlling dissolved O_2_ level. The chemostat operation was activated again only when the biomass concentration was reached 8 g·L^−1^. Chemostat cultivation of *Galdieria sulphuraria* produced over 4 g·L^−1^ of biomass and 60% w/w protein content under both autotrophic and mixotrophic conditions (Abiusi et al. 2022).

## Challenges of scaling-up, economic feasibility and industrial challenges for commercial-scale production

### Scaling-up challenges

Transition from laboratory-scale to industrial-scale production of proteins from microalgae presents a number of technical challenges. Large-scale cultivation under controlled conditions is resource-intensive and requires optimization to achieve consistency in the biomass production and protein content. Biomass generation at industrial-scale remains economically challenging due to a number of constraints. Major limitations for open system cultivation includes (a) poor light utilization by cells, (b) water losses by evaporation, (c) less diffusion of CO_2_ from atmosphere, (d) uncontrolled temperature and (e) requirement of large land-area. Variation in biomass composition in batch-wise due to seasonal changes and cultivation conditions may affect protein yield from microalgae, posing challenges for industrial standardization (Deprá et al. 2025). Closed systems, also known as photobioreactors are engineered with controlled operating systems, which reduce the several limitations of algal growth and problems associated with open-pond systems, such as evaporation of water and contamination with other microbial species. However, algal biomass production could be significantly improved by application of photobioreactor, the major limiting factors are high capital, operation and maintenance costs (Nahdliah et al. 2026). Photosynthetic efficiency and biomass productivity of microalgae are associated with the performance of photobioreactors, which is strongly influenced by reactor geometry, mixing efficiency of gasses in the liquid culture medium and light distribution. Therefore, maintaining optimal operating conditions during high-density cultivation are considerable engineering challenges that require careful process control (Nedaei and Shokrkar 2025). Techno-economic analyses have demonstrated that although microalgae can be cultivated in wastewater with minimal cultivation medium costs, the downstream processing to isolate proteins from microalgae is cost-intensive, thereby increasing the overall production cost of proteins from microalgae. For instance, downstream processing, specifically harvesting, accounts for approximately 20–30% of the total cost of protein production from microalgae (Fasaei et al. 2018). The complex cell-walls of many species of microalgae demand energy-intensive extraction processes, such as mechanical, chemical and enzymatic disruption methods. The limitations of each approach have been reported. Mechanical methods, such as high-pressure homogenization and bead milling can lead to the denaturation of proteins due to the generated heat by mechanical forces. Organic solvent hexane is frequently used in chemical disruption methods, which can pose rising concerns over health and environmental impact (Prasad et al. 2022). Supercritical fluid extraction and ionic liquid-based extraction techniques are emerging, but their high cost limits their industrial adaption (Khalid et al. 2025). The purification of protein extracts to achieve food-grade standard often requires multiple separation steps, thereby increasing the overall processing costs and energy consumption (Kopilovic et al. 2023). Furthermore, industrial-scale protein production from microalgae often struggles to remain economically competitive against traditional crop-based protein sources (Chitolina et al. 2026).

### Economic feasibility

Current microalgal protein production generally requires higher capital investment and operational costs than conventional agricultural- and animal-based protein production. Since protein is usually considered a relatively low-value bulk commodity item, the marketable competitiveness of microalgal protein cannot rely exclusively on the production of protein. Recently published techno-economic assessments indicate that their cost could be reduced in several ways. Future economic viability of the protein production from microalgae will likely depend on the adoption of innovative and energy-efficient pre-treatment technologies and the integration of biorefinery models that maximize biomass utilization (Slegers et al. 2020). In addition, the transition toward fractionation and recovery of high-value compounds, such as secondary metabolites, pigments and lipids along with proteins significantly enhance the overall economic return of the operation. This approach diversifies the product portfolio while minimizing waste, thereby transforming conventional process streams to profitable long-term industrial streams (Kruger et al. 2022). Furthermore, implementation of process intensification strategies, large-scale cultivation technologies and improved downstream processing are projected to further minimize production costs, allowing microalgal proteins to become progressively competitive with conventional animal and plant protein sources in high-valued food and feed markets. However, proteins from microalgae may not yet be economically competitive with conventional bulk protein commodities, they have a considerable market potential in premium applications where nutritional quality and techno-functional properties provide additional value (Sarkar et al. 2026). Nevertheless, the utilization of wastewater as a cultivation medium for the production of proteins using microalgae remains constrained by regulatory concerns. The presence of pathogens and other potentially hazardous compounds (organic compounds and heavy metals) in the wastewater may remain a critical barrier to the application of wastewater for the cultivation medium of microalgae with the aid of protein production. Microalgae can adsorb and accumulate hazardous compounds from wastewater, thereby their intracellular accumulation may trigger the safety and regulatory aspects of microalgal biomass as a protein source (Markou et al. 2018; Álvarez-González et al. 2023). Mechanical methods, such as high-pressure homogenization and bead milling achieve high disruption efficiencies but incur substantial energy costs, while non-mechanical processes like enzymatic hydrolysis, pulsed electric field, and freeze-thaw cycling offer milder conditions but are limited by lower extraction efficiencies and longer processing times (Chen et al. 2025). Saline microalgae-based protein production through greenhouse gas could reduce the production costs by approximately US$80–200 per tonne when compared with high-value co-products, such as whey protein concentrate (Zhang et al. 2025). Furthermore, continued improvements in strain engineering along with implementing advanced cultivation technologies, harvesting methods, downstream processing and integrating biorefinery concept may reduce the limitations of protein production from microalgae. These advancements could improve the economical feasibility and enhance the commercial competitiveness of microalgal protein productions in the global alternative protein market (Alzahmi et al. 2024).

### Industrial challenges

One of the primary challenges is the optimization of cultivation systems and bioreactor operational strategies. Large-scale cultivation requires precise control of environmental parameters, including light intensity, light-dark cycles, nutrient availability, carbon (inorganic and organic) supply, temperature and pH of the cultivation medium, to maximize biomass productivity and protein accumulation. Open pond systems are more susceptible to environmental fluctuations and microbial contamination; whereas, in photobioreactors (closed cultivation system), non-uniform light distribution and self-shading effects reduce photosynthetic efficiency as cell density increases (Barboza-Rodríguez et al. 2024).

Another important challenge for the industrial-scale production of protein is the selection and development of suitable microalgal strain. Different species of microalgae exhibit considerable variation in growth rate, protein content, the composition of amino acids and stress tolerance. Therefore, identifying robust strains which have high biomass productivity and offer superior protein yields is essential for the commercial production of protein by microalgae. Advances in metabolic engineering and system biology have further allowed the development of genetically engineered strains of microalgae with enhanced photosynthetic efficiency, improved nitrogen utilization, increased protein biosynthesis capacity and able to grow under environmental stresses. However, the large-scale production of protein by engineered microalgae remains constrained by regulatory approval, biosafety concerns and the acceptance of consumers (Kselíková et al. 2022).

Downstream processing exhibits another major bottleneck for the commercialization of proteins from microalgae. The technical challenges associated with the selection of efficient solvents, their recovery, cost, safety and regulatory framework**s**. Furthermore, integrating automated process control systems to operate and optimize downstream fractionation could increase energy expenditure (Show et al. 2022). Recent advances in extraction technologies, including pulsed electric fields-, ultrasound-, microwave- and enzyme-assisted extraction could significantly improve the protein extraction from the biomass of microalgae (Nunes et al. 2024); however, their commercial feasibility is debating issue because of significant higher amount of energy consumption (Nitsos et al. 2020).

Regulatory and safety concerns remain considerable major obstakels for the commercialization of proteins derived from microalgae, particularly for food and dietary supplement practices. Concerns regarding the safety, allergenicity and the bioavailability of amino acids of microalgal protein have resulted in stringent regulatory frameworks that hinder their commercialization despite their promising nutritional values (van der Spiegel et al. 2013). Although several species of microalgae have a long history of safe consumption, many novel species and the characteristics of proteins from them still require authorization under regulatory frameworks prior to their commercialization. These regulatory requirements prolong the approval process, increase development costs for producers and other stakeholders, and upsurge market competitiveness, thereby ultimately delaying the large-scale production of proteins from microalgae and their commercialization (Tenzin et al. 2026).

## Conclusion

The rising global adoption of vegetarian and vegan diets across populations has intensified the demand for sustainable and high-quality protein sources. Protein remains a fundamental nutrient for human health; however, conventional animal- and plant-based protein sources present several limitations, including limited availability of arable land, allergenicity, variability in amino acid composition and digestibility. Microalgae have received a great attention for the production of sustainable protein, owing to their rapid growth across a wide range of environmental and trophic conditions, their metabolic flexibility to switch growth modes depending on nutrient availability, their ability to grow in wastewater or non-potable water with minimal nutrient inputs and their exceptional metabolic capabilities. Certain microalgal species contain up to 70% protein by dry weight and provide a nutritionally balanced profile of EAA comparable to traditional protein sources, such as milk, meat, eggs and legumes.

Several critical limitations remain for the industrial expansion of microalgal protein production; however, numerous review articles have addressed the effects of the composition of cultivation medium and operational parameters to obtain high protein yield from microalgae. Therefore, different strategies for process intensification have been emphasized. To intensify the production of proteins from microalgae, strategies such as strain development, the use of cold-adapted microalgae and implying hybrid cultivation system have been taken into consideration. Advances in genome sequencing, genome editing and system-level omics approaches offer innovative opportunities for developing microalgal strains with improved protein biosynthesis pathways. Microalgae can be mutagenized or genetically engineered to express high-quality recombinant proteins with therapeutic importance. It could bring economic upliftment together with the commercialization of conventional microalgal proteins, which aligns with the concept of process intensification. Comprehensive understanding of individual operational parameters forms the underpinning for achieving scalable, stable and economically feasible protein production from microalgae. Therefore, the successful implementation of process intensification strategies requires a systematic understanding of microalgal physiology and its response to different operational parameters. Optimizing key operational parameters, such as light intensity and photoperiod, temperature, nutrient availability (inorganic and organic carbon supply and nitrogen bioavailability), plays a critical role in intensifying microalgal bioprocesses for the biosynthesis of proteins. Hybrid approaches, such as switching of nitrogen-rich cultivation medium from nitrogen-deficient medium, adopting sequential heterotrophic-autotrophic systems and mixotrophic cultivation systems are considered promising strategies for improving microalgal biomass and protein yield. However, protein extraction from microalgae remains technologically challenging due to the presence of structurally complex cell-wall polysaccharides, which hinder efficient protein recovery from the biomass of microalgae. From a process intensification perspective, improving downstream processing systems is equally important to ensure that the enhanced productivity from upstream processes can be translated into economically viable protein recovery. Thus, the development of robust, scalable and cost-effective extraction technologies is essential for the commercialization of microalgal proteins. Nonetheless, effective industrial execution also depends on overcoming several engineering and techno-economic contests, including the design of efficient large-scale cultivation systems, the optimization of process parameters of photobioreactor, the reduction of energy consumption during harvesting and downstream processing, and the integration of economically feasible biorefinery concepts. Furthermore, maintaining consistent biomass productivity and the reliable quality of protein (the composition of amino acid) by large-scale cultivation system while minimizing overall production costs remains a major challenging issue for the commercial accomplishment of microalgal protein production.

One of the biggest gaps in the current scientific literature is that in most investigations only quantify changes in crude protein productivity by microalgae due to the advanced bioprocess and protein nutritional quality (Digestible Indispensable Amino Acid Score (DIAAS), Protein Digestibility-Corrected Amino Acid Score (PDCAAS), essential amino acid index and limiting amino acids) of whole cell microalgae, while comparatively few investigations have addressed how cultivation strategies modulate the overall amino acid profile and protein nutritional quality. Comprehending the molecular mechanisms linking cultivation strategy and associated operating parameters and amino acid biosynthesis remains an imperative research priority for producing nutritionally optimized microalgal proteins.

Microalgal proteins epitomize an unconventional yet highly promising class of sustainable biomolecules in food, feed, cosmeceuticals and pharmaceuticals sectors. It may be realized that process intensification strategies will play a pivotal role in uplifting of protein production from microalgae as a feasible constituent of the global nutrition supply chain, thereby contributing to nutrition security and meeting the rising demand of high-quality, sustainable protein source. Legal frameworks have been consistently considered as a bottleneck for microalgae stakeholders’, regardless of its added-value metabolites and nutritional value of whole biomass. Sustainable commercial production strategy of proteins from microalgae, and comprehensive in *vivo*, clinical and toxicological evaluations are prerequisites before microalgal proteins can be integrated into biopharmaceutical and functional food formulations. Furthermore, harmonized regulatory frameworks, standardized safety and quality assessment protocols, and proper authorized commercialization guidelines for mutagenized and genetically engineered microalgae are indispensable to facilitate the industrial adoption of microalgal protein production and improve consumer confidence in microalgal protein. Considering microalgal proteins in the functional food sector encompasses involvement and coordinated efforts among scientists, industry stakeholders and policymakers. Therefore, future investigations should address advances in strain engineering for the higher protein yield, process intensification strategies for the cultivation of microalgae, downstream processing and producing nutritionally optimized microalgal proteins together with techno-economic and life-cycle assessment and regulatory compliance to establish a commercially sustainable microalgal protein production system.

## Data Availability

Not applicable. Previously published datas are mentioned in this review article, which are cited within the manuscript.
